# Growth in diagnosis and treatment of primary immunodeficiency within the global Jeffrey Modell Centers Network

**DOI:** 10.1186/s13223-022-00662-6

**Published:** 2022-03-04

**Authors:** Jessica Quinn, Vicki Modell, Jordan S. Orange, Fred Modell

**Affiliations:** Jeffrey Modell Foundation, 780 Third Avenue, 47th Floor, New York, NY 10017 USA

**Keywords:** Primary immunodeficiency (PI), Jeffrey Modell Foundation (JMF), Jeffrey Modell Centers Network (JMCN), Network, Awareness, Education, Diagnosis, Treatment, Immunology

## Abstract

**Background:**

Primary immunodeficiencies (PI), which include more than 450 single-gene inborn errors of immunity and may affect up to 1% of the population, are genetic disorders that impair the immune system**.** If not properly identified and treated, individuals with PI are subject to serious, prolonged, and sometimes life-threatening infections or autoimmunity. Despite advancements, awareness of PI remains a critical issue for physicians and the public alike, as this leads to the enhanced and expedited management of these conditions. To address this critical issue, the Jeffrey Modell Foundation (JMF) formed a global network of specialized centers. The goal of this endeavor was to raise awareness of PI to better identify, diagnose, and treat patients, reducing associated mortality and morbidity and improving quality of life (QOL). For more than two decades, the Jeffrey Modell Centers Network (JMCN) has served as the foundation upon which these goals have been pursued. The JMCN currently includes 909 Expert Physicians at 400 institutions, in 316 cities, and 86 countries spanning six continents.

**Methods:**

A survey was developed by JMF for members of the JMCN, following the most recent Classification of PI from the IUIS Expert Committee, to periodically describe the patient population, including treatment modalities and demographics. Physician-reported data from 2021 was compared to that from 2018 and 2013. Physicians in the JMCN also reported on select outcomes of their PI patients one year prior to and one year following diagnosis.

**Results:**

A total of 300 JMF Physician Surveys from 681 physicians were included in this analysis. This is a 75% physician response rate. From 2013 to 2021, there was a 96.3% increase in patients followed in the US and an 86.1% increase globally. During the same period, patients identified with a specific PI defect increased by 46.6% in the US and 47.9% globally. Patients receiving IgG and HSCT increased by 110% and 201% respectfully since 2013. Early diagnosis led to reported decreased morbidity and mortality and reduced calculated healthcare costs.

**Conclusions:**

This global analysis of physician-reported data on patients with PI demonstrates an increase in both diagnosed and treated patients. This substantial increase from within the JMCN is a testament to its impact. In addition to building an extensive global patient database, the expanding JMCN serves as a unique and critical resource, providing the infrastructure for earliest diagnosis, optimized treatments, and implementation of standard-of-care and best practices. The JMCN provides a critical platform that facilitates the education of physicians and patients, awareness initiatives, and research advances, through collaboration and connectivity, ultimately resulting in improved outcomes and QOL for patients with PI. The JMCN has steadily and substantially grown for more than two decades and continues to substantively impact the field of Immunology globally.

**Supplementary Information:**

The online version contains supplementary material available at 10.1186/s13223-022-00662-6.

## Key messages

1. The JMCN has steadily and substantially grown for more than 2 decades and continues to significantly influence the global Immunology community.
2. In addition to compiling an extensive global patient database, the expanding JMCN acts as a unique and critical resource, increasing awareness of PIs, educating physicians and patients, and facilitating diagnosis via expertise and genetic sequencing, as well as treatment.
3. The JMCN provides a platform to optimize research advances, earliest diagnosis, enhanced treatments, and implement standard-of-care and best practices through maximized connectivity, resulting in a better quality of life and improved outcomes for patients with PI.

**Supplementary Information:**

The online version contains supplementary material available at 10.1186/s13223-022-00662-6.

## Introduction

Primary immunodeficiencies (PI), which include over 450 single-gene inborn errors of immunity (IEI), are genetic disorders that impair the immune system [1–3]. Individuals with PI are subject to serious, prolonged, and sometimes life-threatening infections and autoimmunity when not properly identified and treated [1, 4–7]. Manifestations of PI can range from life-threatening, such as Severe Combined Immunodeficiency (SCID), to vulnerability to common and opportunistic infections, persistent inflammation, and autoimmunity. Up to 1% of the global population may have a PI when all IEIs are considered, which is more than previously predicted [2, 8, 9].

Despite advancements in research, genetic sequencing, molecular diagnosis, and treatments that increased our grasp of the immune system and bettered quality of life (QOL) for individuals with PI, awareness of PI remains a critical issue for physicians and the public alike. Increasing awareness of PI leads to the enhanced and expedited management of these conditions [10–16].

**Fig. 1:**
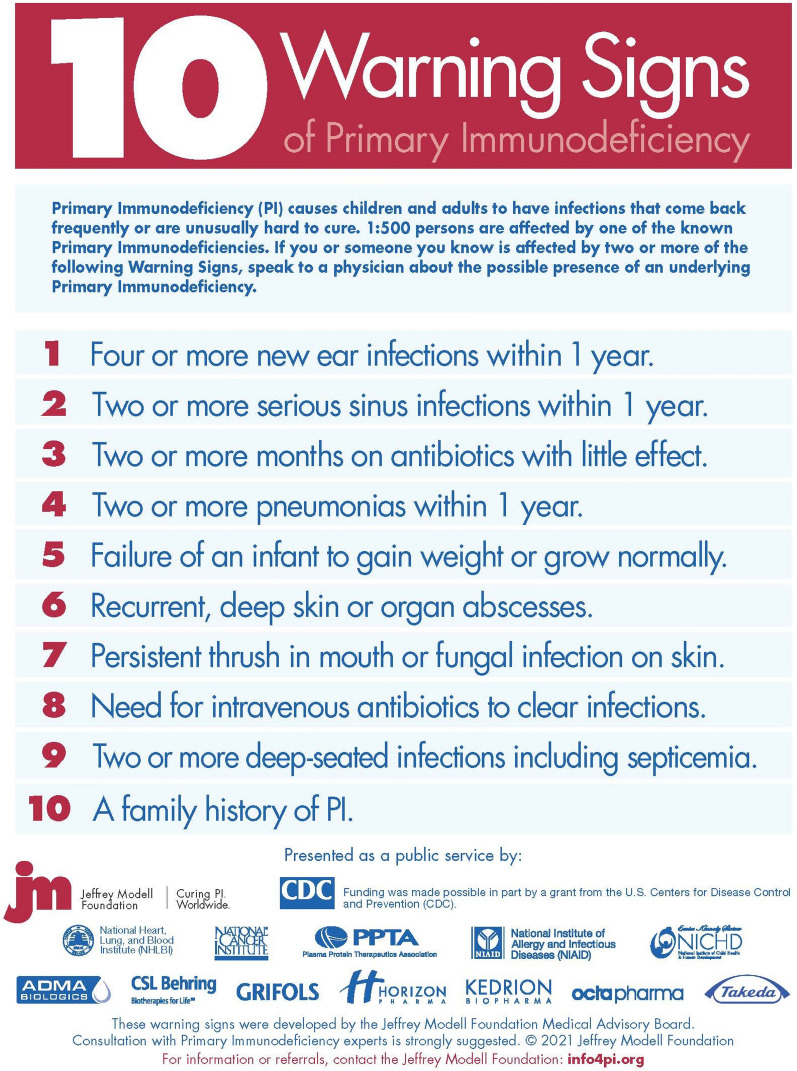
JMF’s 10 warning signs of PI

**Fig. 2:**
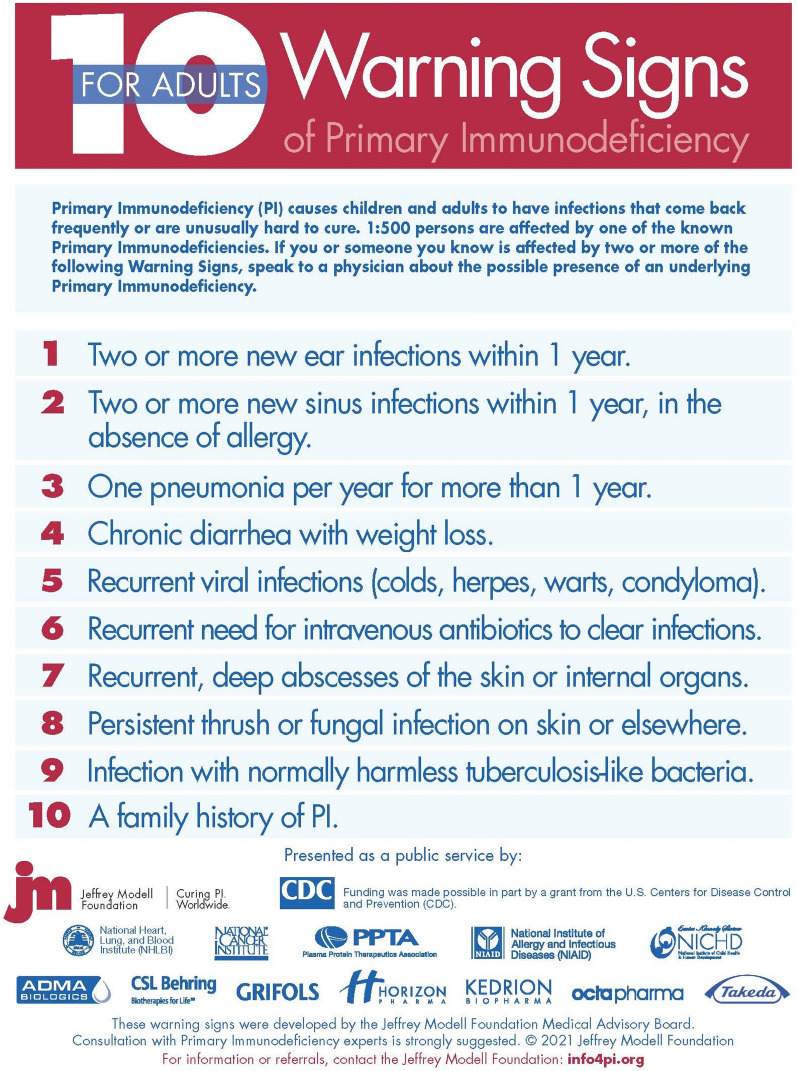
JMF’s adult 10 warning signs of PI

To address this critical issue, the Jeffrey Modell Foundation (JMF) formed a global network of specialized centers and developed JMF’s 10 Warning Signs of PI (Figs. 1, 2). The goal of this endeavor was to raise awareness of PI to better identify, diagnose, and treat patients, provide tools useful in these endeavors, and ultimately reduce associated mortality and morbidity and improve QOL. For more than two decades, the Jeffrey Modell Centers Network (JMCN) has laid the groundwork to accomplish these goals, with 909 Expert Physicians at 400 institutions, in 316 cities and 86 countries spanning six continents. A list of expert physicians in the JMCN is shared, with permission, on JMF’s website through the “Find an Expert” tool, which can be accessed at http://info4pi.org/information-booth/find-an-expert. The network is continually expanding, with growth each year, further highlighting its influence over the past two decades as a sought-after resource for the PI community.

A detailed survey for members of the JMCN was developed by JMF, following the most recent Classification of PI from the International Union of Immunological Societies (IUIS) Expert Committee [17, 18], to periodically report on the PI patient population, including treatments utilized and demographics (Additional file 1: Figure S1). Additional objectives of the survey were to provide meaningful information to quantify and evaluate the effectiveness and ultimate health impact of JMF’s awareness and advocacy activities, to assess the growth of the patient population, to assess treatment modalities, to identify any notable changes or patterns, and to provide periodic comprehensive reports to the JMCN and broader PI community. The IUIS classification is an invaluable resource for immunologists and researchers everywhere and currently includes 416 distinct disorders with nearly 450 different gene defects listed [17]. In the past two years, 64 new gene defects were either discovered or confirmed [17], due in part to the increased use of exome sequencing (ES) and next generation sequencing (NGS). Several of these new IEI have been found in either an individual or a just a few kindreds, which may not offer a comprehensive description of prevalence and phenotype [17]. Further complicating accurate diagnosis, is the considerable expansion of phenotypes associated with specific diseases and gene variants of uncertain significance, both associated with the increased use and accessibility of NGS [17].

## Methods

### Growth of the JMCN

The JMCN is currently made up of 909 expert physicians at 400 institutions, in 316 cities, and 86 countries across 6 continents, and is always growing as physicians seek to be included. A comprehensive catalog of all JMCN members and their affiliated centers is maintained and updated on a regular basis, as expert immunologists join or leave the network (due to retirement, relocation, change in employment, etc.). The current make-up of the JMCN was compared to that from 2018 and 2013, which has been previously published [19, 20].

### JMF Physician Survey

The JMF Physician Survey follows the most recent version of the Classification of PI from the IUIS Expert Committee available at the time [17, 18] (and before the most recent interim update) [3] and lists the Online Mendelian Inheritance in Man (OMIM) number for each defect or gene, if available. When appropriate, the inheritance and mutation type is provided for each gene, as per the IUIS document.

The 2020–2021 survey, which adheres to the latest IUIS classification tables, requested information on patients followed and diagnosed with a PI. A section was provided to list any genes or disorders not listed in the survey tables. The survey also requested information on the administration of immunoglobulin therapies, hematopoietic stem cell transplantation (HSCT), and gene therapy. Additionally, a demographics section was provided requesting information regarding gender and age.

Surveys were sent via email to the entire JMCN, requesting information in 2020–2021, and were sent back to JMF by email or fax. Importantly, all information provided was HIPAA compliant and no identifying information was obtained. The 2021 physician-reported data was then compared to that from 2018 and 2013, which was obtained in a similar manner and format and has been previously published [19, 20].

### Clinical outcomes and cost analysis

We asked physicians in the JMCN to report on outcomes of their PI patients one year prior to and one year following diagnosis including the number of episodes of specific infections, days in the hospital, ER and office visits, days on medication, and missed school or workdays. These findings should be considered as physician-reported and directional since validation of outcomes was not mandatory.

The cost per episode for specific conditions was determined using the Agency for Healthcare Research Quality (AHRQ) Medical Expenditure Panel Survey (MEPS), which provides data on health care utilization and expenditures by medical condition [21]. The cost of hospitalization per day was obtained from the Kaiser Family Foundation report on the American Hospital Association (AHA) annual survey [22]. The associated costs of physician visits, emergency room visits, and antibiotic treatment were acquired from the Health Care Cost Institute (HCCI) 2018 Health Care Cost and Utilization Report, which utilizes data from 40 million enrollees of employer-sponsored health insurance annually between 2014 and 2018 [23]. The costs associated with missed days of work were derived from the United States Social Security Administration [24]. Additional and supporting data were obtained from the Hospital Cost and Utilization Project (HCUP) [25]. HCUP offers the largest inpatient care database in the US, including more than 7 million hospital stays, allowing for examination of special populations, uncommon treatments, and rare conditions [25]. Given the sources used, however, it should be assumed that the costs estimated are more specific to those found in the US and may be distinct from those encountered in other countries.

### Statistical analysis

Responses were recorded in a secure Microsoft Excel spreadsheet, with quality control measures in place to maximize the quality of data entry. Data fields corresponded with those of the 2020–2021 survey (Additional file 1: Figure S1). Descriptive quantitative statistical analyses were performed to summarize the responses including the distribution, percent change, prevalence, and mean, each when appropriate. Differences between select geographical areas were examined, as well as between select years. Regarding missing values, there were many ranging from 0 to 97.3% (Additional file 2: Table S1), depending upon field and question, based on 300 surveys received (n = 300). Due to the survey design and instructions, for all fields other than demographics, a missing response was interpreted as a “zero” in that the physician did not have any patients having that particular condition or receiving that particular treatment. For the demographic fields, analysis was performed only for the available data, as the missing values do not relate to any of the other data fields.

## Results

### Growth of the JMCN

**Fig. 3:**
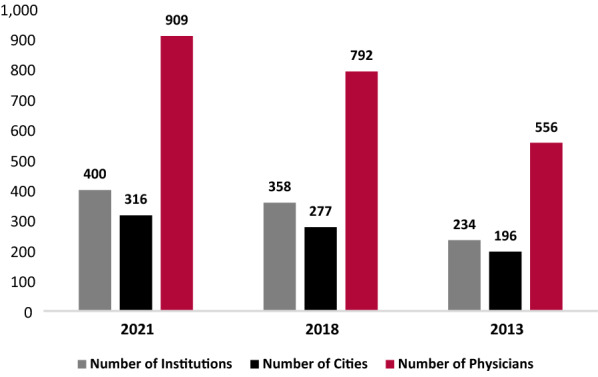
Growth of the Jeffrey Modell Centers Network (JMCN)

**Fig. 4:**
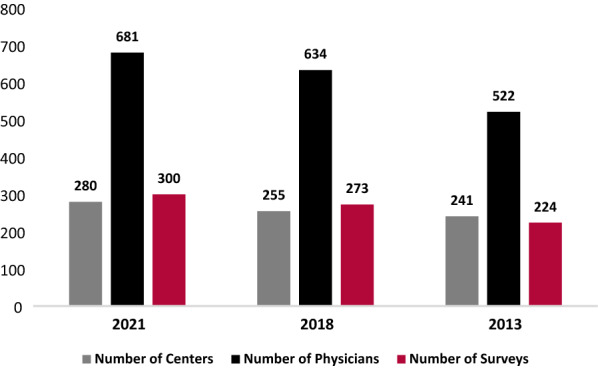
Reports received from the JMCN

The JMCN is made up of 909 expert physicians at 400 institutions, in 316 cities, and 86 countries across 6 continents. Since 2013, the number of institutions, cities, and physicians included in the network have increased by 70.9%, 61.2%, and 63.5%, respectively (Fig. 3). A total of 300 JMF Physician Surveys from 681 physicians were included in this analysis, representing 80 countries, 227 cities, and 280 institutions. This is a 75% physician response rate. Nearly 30% of reporting physicians were located in the US, representing 32 states. The number of reporting Centers, reporting physicians, and surveys received increased by 16.2%, 30.5%, and 33.9% respectively from 2013 to 2021 (Fig. 4). Thus, at a minimum, the increase in any category between 2013 and 2021 that was over 34% is unlikely to have resulted from simply an increase in responses. That said, since most of the largest centers were part of the JMCN in 2013 that percentage expectation should be considered conservative.

### Prevalence

**Fig. 5:**
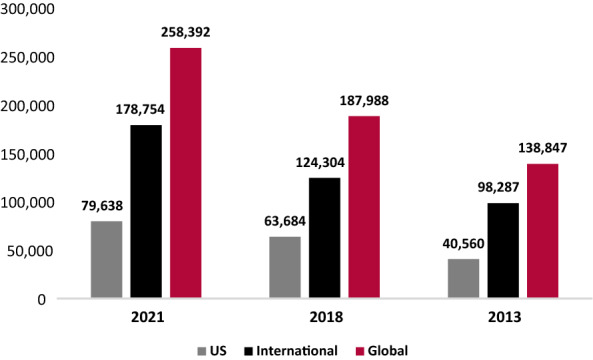
Physician-reported prevalence of PI: patients followed

**Fig. 6:**
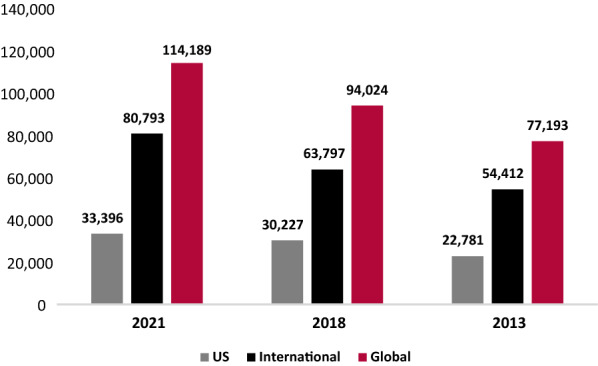
Physician-reported prevalence of PI: patients identified with PI defects

Physician-reported prevalence of patients with PI increased from 2013 to 2021, with a 96.3% increase in patients followed in the US and an 86.1% increase globally (Fig. 5). During the same period, patients identified with a specific PI defect increased by 46.6% in the US and 47.9% globally (Fig. 6). “International” includes all reports except the US, while “Global” includes all reports. Given an increase of 16.2% in sites reporting during this period, the increase in prevalence and other reported measures were unlikely due to an increase in reporting centers alone, and so the data presented were not normalized.

From 2013 to 2021, total patients followed, and total patients identified with a specific PI defect were compared across nine geographical regions. Total patients followed increased across all nine geographical regions, with an increase of 34.1% in Canada, 128% in Latin America, 140.5% in Western Europe, 28.9% in Eastern Europe, 78% in the Middle East, 53.5% in Asia, 2,073.6% in Australia, and 98.6% in Africa (Table 1). Total patients identified with a specific PI defect increased by 77.8% in Latin America, 45.2% in Western Europe, 25.7% in Eastern Europe, 92.4% in the Middle East, 128.5% in Asia, 22.5% in Africa, and 1,873.6% in Australia (Table 1). Importantly, during this period, there was a substantial increase in reporting sites in Latin America, but this only explains a fraction of the growth in patients. In the US, Canada, Western Europe, Eastern Europe, Asia, and Australia there was a slight increase in reporting sites. In the Middle East, there was a minor decrease in reporting sites, while in Africa, reporting sites remained stable.

**Table 1:** Physician-reported prevalence of PI by region

	Patients followed				Patients identified w/PI defects			
	2021	2018	2013	% Change (%)	2021	2018	2013	% change (%)
US	79,638	63,684	40,560	96.3	33,396	30,227	22,781	46.6
Canada	5440	4923	4058	34.1	3078	3047	3880	− 20.7
Latin America	12,257	12,487	5377	128.0	9531	8793	5361	77.8
Western Europe	86,399	46,011	35,932	140.5	37,040	28,592	25,518	45.2
Eastern Europe	54,743	47,525	42,458	28.9	14,936	11,631	11,886	25.7
Middle East	9823	7155	5520	78.0	8408	5664	4370	92.4
Asia	5178	2581	3373	53.5	4212	2358	1843	128.5
Australia	1978	1927	91	2073.6	1796	1876	91	1873.6
Africa	2936	1695	1478	98.6	1792	1836	1463	22.5
Total	258,392	187,988	138,847	86.1	114,189	94,024	77,193	47.9

The number of patients followed and identified with a specific PI defect by region in 2021, 2018, and 2013

### IUIS classification

**Fig. 7:**
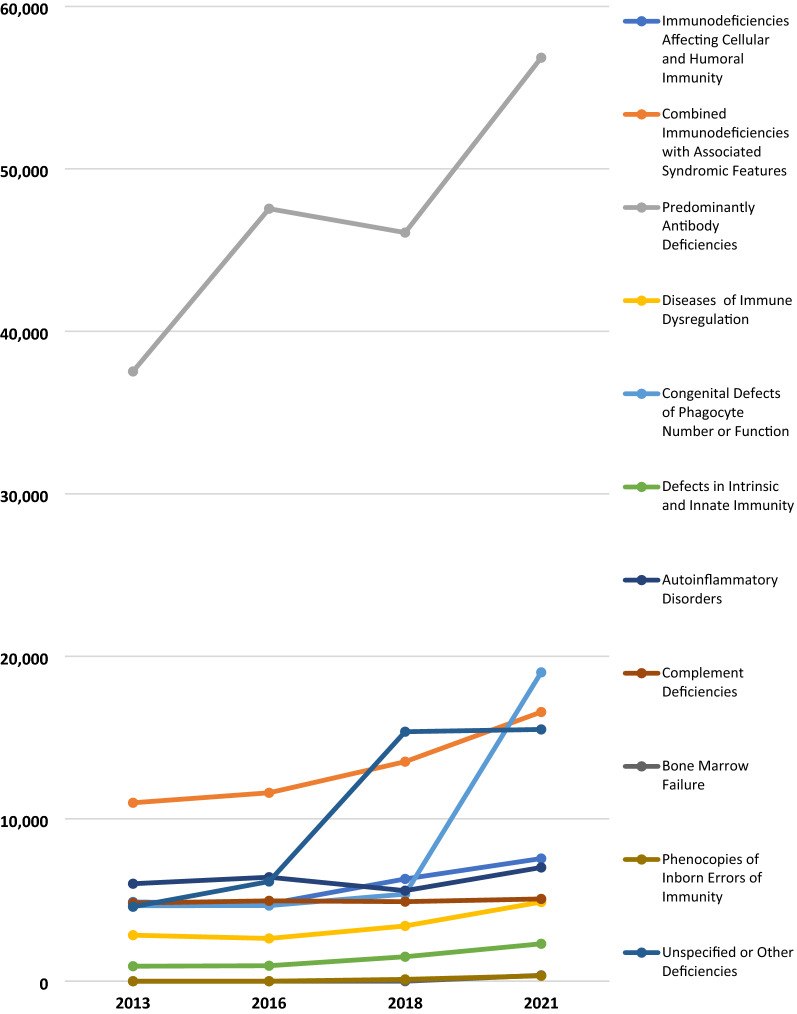
Physician-reported prevalence of PI by IUIS classification, 2013–2021

The 2021 patient distribution according to the IUIS classification of PI categories was examined in the US, internationally, and globally. Physicians reported that Predominantly Antibody Deficiencies accounted for 56% of patients with a specific PI defect in the US, 37% internationally, and 42% globally (Table 2). Combined Immunodeficiencies with Associated Syndromic Features accounted for 17% of patients with a specific PI defect in the US, 11% internationally, and 12% globally. Unspecified or Other Deficiencies accounted for 9% of these patients in the US, 12% internationally, and 11% globally; a 4% reduction since 2018. Figure 7 shows the change in physician-reported prevalence of PI by the IUIS diagnostic category from 2013 to 2021.

**Table 2:** Physician-reported prevalence of PI by classification, 2021

	US	INT’L	Global
Immunodeficiencies affecting cellular and humoral immunity	1566	5985	7551
Combined immunodeficiencies with associated syndromic features	5677	10,892	16,569
Predominantly antibody deficiencies	19,423	37,418	56,841
Diseases of immune dysregulation	1199	3676	4875
Congenital defects of phagocyte number or function	1678	17,332	19,010
Defects in intrinsic and innate immunity	311	1993	2304
Autoinflammatory disorders	673	6328	7001
Complement deficiencies	646	4418	5064
Bone marrow failure	58	306	364
Phenocopies of inborn errors of immunity	48	286	334
Unspecified or other deficiencies	3135	12,362	15,497
Total^a^	34,414	100,996	135,410

The 2021 distribution of patients according to the IUIS classification of PI categories

^a^Global total doesn’t match Table 1 due to the inclusion of “Unspecified or Other Deficiencies” and reporting

**Fig. 8:**
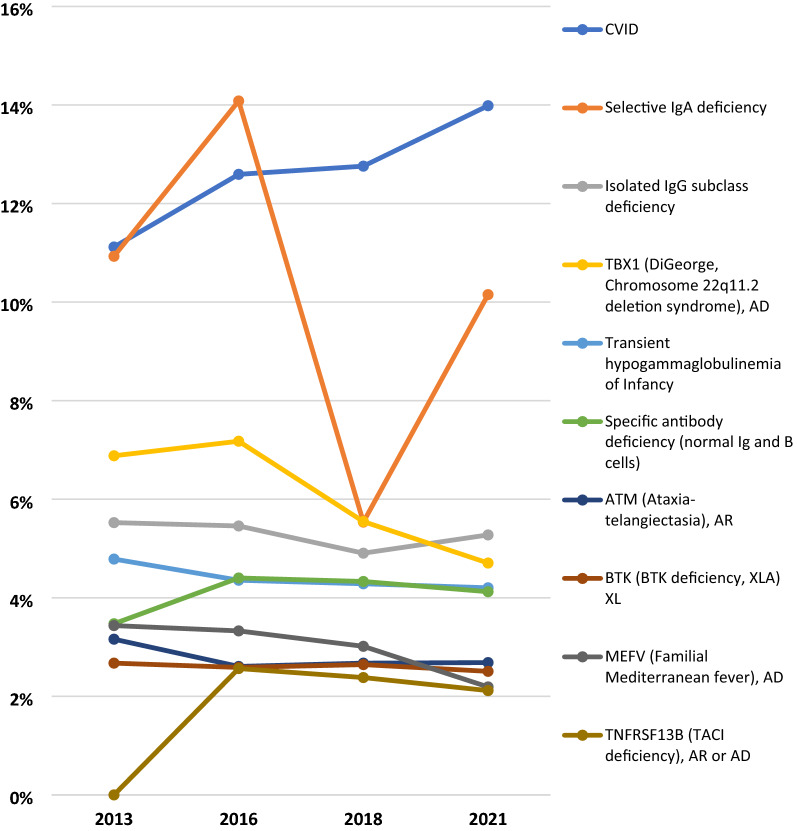
Physician-reported prevalence of the 10 most commonly identified PI defects, 2013–2021

The fifteen most prevalent PI defects were examined by geographical region. The most prevalent was Common Variable Immunodeficiency (CVID), with 16.6% in the US, 12.9% internationally, and 13.9% globally (Table 3). Specifically, for CVID, Canada had a 22.5% prevalence while Australia had a 34.2% prevalence. There was a prevalence of 10.8% for Selective IgA Deficiency in the US, 9.9% internationally, and 10.2% globally. The Middle East reported a prevalence of 19.2% Familial Mediterranean Fever compared to a 2.2% prevalence globally, and Africa reported a 7% prevalence of Ataxia Telangiectasia compared to a 2.7% prevalence globally. The fifteen most prevalent PI defects have remained quite consistent over the past eight years, with only minor differences, potentially due to modifications in classification. Figure 8 shows the change in physician-reported prevalence of 10 PI defects from 2013 to 2021.

**Table 3:** Physician-reported prevalence of 15 PI defects by region, 2021 report

		US	Canada	Latin America	West Europe	East Europe	Middle East	Asia	Australia	Africa	Global total
1	CVID, unknown	5551	694	1191	5592	1100	753	312	615	161	15,969
2	Selective IgA deficiency	3596	63	1550	3577	2298	320	71	94	23	11,592
3	Isolated IgG subclass deficiency	986	13	136	4,105	333	165	30	240	16	6024
4	TBX1 (DiGeorge, Chromosome 22q11.2 deletion syndrome), AD	2,384	150	325	1560	614	105	170	37	28	5373
5	Transient hypogammaglobulinemia of infancy	900	136	602	437	2500	118	56	9	41	4799
6	Specific antibody deficiency (normal Ig and B cells)	3029	119	589	744	74	66	32	46	9	4708
7	ATM (Ataxia-telangiectasia), AR	1262	63	267	528	487	255	61	17	126	3066
8	BTK (BTK deficiency, XLA) XL	527	101	361	811	342	170	375	116	60	2863
9	MEFV (Familial Mediterranean fever), AD	223	18	31	441	90	1612	23	32	32	2502
10	TNFRSF13B (TACI deficiency), AR or AD	2122	5	18	223	20	9	14	3	3	2417
11	DiGeorge Syndrome, unknown	988	103	135	347	301	150	85	12	41	2162
12	C1QA (C1q deficiency), AR	167	27	133	1264	177	29	10	0	11	1818
13	CYBB (X-linked CGD (gp91 phox)), XL	356	45	211	441	202	63	378	17	39	1752
14	IgG subclass deficiency with IgA deficiency	574	73	84	691	149	52	30	0	20	1673
15	WAS [Wiskott-Aldrich syndrome (WAS LOF)], XL	282	49	181	455	215	69	237	26	23	1537
Total		22,947	1659	5814	21,216	8902	3936	1884	1264	633	68,255

The distribution of the 15 most commonly identified PI defects globally by region

### Treatment

Immunoglobulin therapies were examined from 2013 to 2021. According to physician reports, since 2013, patients receiving IgG increased by 110% (Table 4). A total of 32% of all patients with a PI defect receive immunoglobulin replacement therapy. Patients receiving subcutaneous immunoglobulin (SCIG) increased by 378% since 2013 and patients getting Intravenous immunoglobulin therapy (IVIG) in the hospital or clinic increased by 114.4%. There was a 22.2% decrease in patients getting IVIG at home during this period, however a 378% increase in patients getting SCIG more than compensates for this decrease.

**Table 4:** Treatment with IgG by site of care

	2021	2018	2013	% change (%)
IVIG—clinic				
US	3604	3299	2572	40.1
INT’L	15,385	9296	6285	144.8
Global	18,989	12,595	8857	114.4
IVIG—home				
US	2449	2381	2423	1.1
INT’L	895	842	418	114.1
Global	3344	3223	4298	− 22.2
SCIG				
US	3822	2881	1631	134.3
INT’L	9759	4778	2667	265.9
Global	13,581	7659	2841	378.0
Total, including “other”				
US	9984	8721	7315	36.5
INT’L	26,186	15,246	9910	164.2
Global	36,170	23,967	17,225	110.0

The number of patients reported to receive immunoglobulin therapy (IgG) intravenously in the clinic and at home, subcutaneously, and by other treatment modalities in 2021, 2018, and 2013

Immunoglobulin therapies in 2021 were examined in nine geographical regions. Notably, 53% of patients treated with IgG in Western Europe receive SCIG compared to 37.5% globally, and 75% of patients getting IgG in Latin America are treated in the clinic or hospital compared to 52.4% globally (Table 5). Additionally, 24.5% of patients getting IgG in the US, do so at home compared to 9.2% globally.

**Table 5:** Treatment with IgG by site of care by region

	US	Canada	Latin America	West Europe	East Europe	Middle East	Asia	Australia	Africa	Global totals
IVIG—clinic	3604	717	2327	5646	2472	1981	703	1042	497	18,989
IVIG—home	2449	11	67	814	0	1	0	1	1	3344
SCIG	3822	523	636	7450	927	24	27	136	36	13,581
Other	109	0	74	47	3	15	0	1	7	256
Totals	9984	1251	3104	13,957	3402	2021	730	1180	541	36,170

The number of patients reported to receive IgG intravenously in the clinic and at home, subcutaneously, and by other treatment modalities by region, 2021

From 2013 to 2021, treatment with immunoglobulin therapy was examined in nine geographical regions. A 110% global rise in the number of patients receiving IgG was seen during this period, even with just a 16.2% increase in reporting centers. However, a rise in reporting Centers in Latin America likely resulted in the substantial growth in patients receiving IgG reported in that region (Table 6).

**Table 6:** Treatment with IgG by region

	2021	2018	2013	% change (%)
US	9984	8721	7315	36.5
Canada	1251	1113	756	65.5
Latin America	3104	1901	851	264.7
Western Europe	13,957	7375	5343	161.2
Eastern Europe	3402	2205	1675	103.1
Middle East	2021	739	485	316.7
Asia	730	391	322	126.7
Australia	1180	1125	21	5519.0
Africa	541	397	457	18.4
Total	36,170	23,967	17,225	110.0

The number of patients reported to receive IgG by region in 2021, 2018, and 2013

In addition to treatment with immunoglobulin therapy, additional treatments were assessed, such as HSCT, PEG-ADA, and gene therapy. There was substantial growth in patients obtaining these treatments for PI, with the most patients receiving HSCT, then gene therapy and PEG-ADA (Table 7). These treatment methods were examined in nine geographical regions in 2021. Gene therapy was reported in the greatest number of patients in Western Europe.

**Table 7:** Other treatments by region

	US	Canada	Latin America	West Europe	East Europe	Middle East	Asia	Australia	Africa	Global totals
Patients treated by transplant	1628	387	373	2808	675	766	290	35	70	7032
Patients treated by gene therapy	89	13	9	119	7	5	2	0	4	248
Patients treated with PEG-ADA	49	8	4	51	10	2	1	0	1	126
Total	1766	408	386	2978	692	773	293	35	75	7406

The number of patients with severe PIs reported to receive gene therapy, PEG-ADA, or a hematopoietic stem cell or thymus transplant by region, 2021

As practices in HSCT have been evolving, greater detail in the use of this treatment was pursued. For patients reported to receive HSCT, stem cell donor type was assessed from 2013 to 2021. The number of patients treated by HSCT increased by 201%. Further, there was a 249% increase in matched donor transplants, a 190% increase in matched unrelated donor transplants, a 183% increase in mismatched unrelated donor transplants, and a 162% increase in parental haplo transplants (Table 8). Stem cell donor type was examined in nine geographical regions in 2021. The most common stem cell donor type globally was matched unrelated, followed by matched related (Table 9). In the Middle East, Africa, and Eastern Europe matched related was the most common stem cell donor type. In Western Europe and Australia, parental haploidentical transplants accounted for 28% of HSCT, markedly more than the other regions.

**Table 8:** Stem cell donor type used for patients having received HSCT

	2021	2018	2013	% change (%)
MRD				
US	290	229	76	281.6
INT’L	1624	1103	472	244.1
Global	1914	1332	548	249.3
MUD				
US	535	362	151	254.3
INT’L	1483	1162	544	172.6
Global	2018	1524	695	190.4
mMUD				
US	160	138	29	451.7
INT’L	302	211	134	125.4
Global	462	349	163	183.4
Parental haplo				
US	218	126	57	282.5
INT'L	906	727	372	143.5
Global	1124	853	429	162.0
Total				
US	1203	855	313	284.3
INT'L	4315	3203	1522	183.5
Global	5518	4058	1835	200.7

The number of patients reported to receive HSCT from matched related donors, matched unrelated donors, mismatched unrelated donors, and parental donors in 2021, 2018, and 2013

**Table 9:** Stem cell donor type used for patients having received HSCT by region

Donor type	US	Canada	Latin America	West Europe	East Europe	Middle East	Asia	Australia	Africa	Global totals
MRD	290	91	108	623	284	398	68	7	45	1914
MUD	535	239	123	666	276	46	100	21	12	2018
mMUD	160	31	13	199	7	30	22	0	0	462
Parental haplo	218	20	83	577	71	79	52	11	13	1124
Totals	1203	381	327	2065	638	553	242	39	70	5518

The number of patients reported to receive HSCT from matched related donors, matched unrelated donors, mismatched unrelated donors, and parental donors by region, 2021

For patients receiving HSCT, the stem cell source was assessed from 2013 to 2021. There was a 224.2% increase in bone marrow as the stem cell source and a 248.7% increase in cord blood (Table 10). Stem cell source in 2021 was examined in nine geographical regions and varied significantly by region. It was reported that 56.9% of transplants in Latin America used bone marrow, compared to 61% globally (Table 11). In the Middle East, 36.3% of transplants used peripheral stem cells, compared to 24.4% globally, and 35.6% of the transplants used cord blood in Asia, compared to 12% globally.

**Table 10:** Stem cell source used for patients having received HSCT

	2021	2018	2013	% change (%)
Bone marrow				
US	794	585	189	320.1
INT’L	2539	1953	839	202.6
Global	3333	2538	1028	224.2
Peripheral stem cells				
US	227	131	53	328.3
INT’L	1111	729	401	177.1
Global	1338	860	454	194.7
Cord blood				
US	151	147	47	221.3
INT’L	515	408	144	257.6
Global	666	555	191	248.7
Total, including “other”				
US	1284	865	290	342.8
INT’L	4180	3097	1392	200.3
Global	5464	3962	1682	224.9

The number of patients reported to receive transplantation through the source of bone marrow, peripheral stem cells, cord blood, or other stem cell sources in 2021, 2018, and 2013

**Table 11:** Stem cell source used for patients having received HSCT by region

Stem cell source	US	Canada	Latin America	West Europe	East Europe	Middle East	Asia	Australia	Africa	Global totals
Bone marrow	794	217	185	1374	307	290	103	30	33	3333
Peripheral stem cells	227	56	62	508	196	190	64	1	34	1338
Cord blood	151	72	78	196	23	42	93	8	3	666
Other	112	1	0	7	6	0	1	0	0	127
Totals	1284	346	325	2085	532	522	261	39	70	5464

The number of patients reported to receive transplantation through the source of bone marrow, peripheral stem cells, cord blood, or other stem cell sources by region, 2021

The substantial growth described above is partially due to newborn screening, molecular diagnosis, and NGS, and may need additional evaluation in the future. Notably, some Centers did not report treatment data, potentially due to access issues, data availability, or specific hospital guidelines.

### Demographics

Physician-reported patient demographics in 2021 were compared with that of 2018 and 2013. Reports included information on gender for 62,045 patients and on age for 56,187 patients (Table 12). Of these patients, 58% were male and 42% were female, globally. In the US alone, 56.3% were male, and 43.7% were female. It was reported that 64.2% of these patients were 0–19 years old, and 35.8% were 20 + years old, globally. The demographic distribution has largely remained consistent since 2013.

**Table 12:** Patient gender and age

	2021			2018			2013	
	US	INT’L	Global	US	INT’L	Global	Global	% change (%)
Gender								
Male	7334	28,627	35,961	6085	19,790	25,875	3540	915.8
Female	5695	20,389	26,084	4763	13,944	18,707	2803	830.6
Total	13,029	49,016	62,045	10,848	33,734	44,582	6343	878.2
Age								
< 1 year	958	3148	4106	672	2211	2883	149	2655.7
1–4 years	2712	7641	10,353	1797	4987	6784		
5–19 years	5196	16,431	21,627	3894	11,366	15,260		
Total pediatric	8866	27,220	36,086	6363	18,564	24,927		
20–39 years	1769	7161	8930	2042	5855	7897		
> 40 years	3287	7884	11,171	2092	4111	6203		
Total adult	5056	15,045	20,101	4134	9966	14,100		
Grand total	13,922	42,265	56,187	10,497	28,530	39,027	5993	837.5

The number of patients by age and gender in the United States and internationally in 2021, 2018, and 2013

### Clinical outcomes and cost analysis

Physicians in the JMCN reported on outcomes of their PI patients 1-year pre and 1 year post diagnosis, including episodes of specific infections, days in the hospital, ER and office visits, days on certain medications, and missed school or workdays. For each condition analyzed, the average number of reported episodes decreased after diagnosis, as did the number of hospital days, ER and office visits, days on antibiotics, and missed school or workdays, resulting in overall decreased morbidity and mortality. Importantly, early diagnosis also reduced estimated healthcare costs (using US cost bases), even with routine IgG replacement therapy (Table 13). Each diagnosed patient translated to a $97,488 annual savings to the healthcare system. The savings remain at $87,888 annually, even for diagnosed patients treated with IgG.

**Table 13:** PI post-diagnosis average annual estimated savings with and without IgG

Condition	Pre Dx average no. of episodes	Post Dx average no. of episodes	Cost per episode	Pre Dx annual cost	Post Dx annual cost	Post Dx average annual savings
Persistent otitis media	4.2	1.6	$607	$2549	$971	$1578
Serious sinus and upper respiratory infections	4.6	2.1	$1125	$5175	$2362	$2813
Viral infections	3.7	1.4	$2038	$7540	$2853	$4687
Acute bronchitis	3.1	0.8	$468	$1450	$374	$1076
Bacterial pneumonias	2.8	0.6	$4748	$13,294	$2848	$10,446
Chronic obstructive pulmonary disease and bronchiectasis	4.3	1.4	$2136	$9184	$2990	$6194
Hospitalization days	19.8	3.1	$2607	$51,618	$8081	$43,537
Physician/ER visits	70.8	11.7	$367	$25,983	$4293	$21,690
Days on antibiotics	166.2	72.8	$5	$831	$364	$467
School/work days missed	33.9	8.9	$200	$6780	$1780	$5000
Total per patient without IgG				$124,404	$26,916	$97,488
Total per patient treated with IgG [impact of IgG treatment weighted for 32% of identified patients in database (average annual cost of IgG ($30,000) × 32%)]				$124,404	$36,516	$87,888

The estimated costs of the most frequent conditions affecting PI patients pre- and post-diagnosis and the post-diagnosis average annual savings with and without IgG

## Discussion

### The JMCN

The JMCN was developed by JMF to meet the rising need for specialized centers to accommodate the increasing patient population and to create the infrastructure needed to promote research, early diagnosis, and proper treatment. Physicians in the JMCN have reported continued growth in the identification, diagnosis, and treatment of patients with PI in the twenty years since the JMCN was established and education and awareness initiatives were introduced. The JMCN is continuously expanding, and patients with PI are increasingly being identified within the network and in general [26], allowing them to receive earlier diagnosis and appropriate treatment, leading to improved outcomes and QOL.

This comprehensive global analysis of physician-reported data on patients with PI demonstrates an increase in diagnosed and treated patients that generally outpaces any overall increase in surveys returned to JMF. The considerable growth in patients can likely be attributed to newborn screening, education and awareness activities, molecular diagnosis, and increased availability of genetic diagnostics including NGS platforms. There continue to be notable regional differences within the JMCN, likely due to founder effects and consanguinity increasing the prevalence of certain defects [15, 27]. This should inform future awareness campaigns, which can be developed to address the unique needs of each geographical region. Efforts, such as continuing medical education, can be focused and tailored to identify risk categories more accurately through further assessment of specific genes. Opportunities for targeted education and resourcing should be enhanced to embolden experts who can truly impact outcomes for patients with PI to hopefully save lives.

Although the reported proportion of patients found with a specific defect has decreased by about 10% over the previous eight years, there was an overall increase in patients reported and a 4% total reduction in patients reported with an unspecified defect, since 2018. The recent less dramatic rise in patients found with a specific defect is likely a consequence of the JMCN continuously expanding into regions with limited to no access to molecular diagnosis and genetic sequencing. Although patients with PI are being identified at an increasingly higher rate and there are meaningful advancements in genomic technologies underway, further improvements need to be made.

Additionally, a limitation of this analysis is the presence of missing responses. To mitigate this limitation and reduce missing responses in the future, serious consideration is being given to transitioning to the use of electronic forms with each field requiring a response within a specified range when appropriate.

### Clinical outcomes and cost analysis

Over the previous few decades, there has been a drastic improvement in patient outcomes across the spectrum of PI diagnoses due to early recognition [28–30]. In addition to the seemingly obvious improvements in outcomes for patients, early recognition of PI results in annual estimated savings to the healthcare system (US-based) for each diagnosed patient of $97,488. Even when accounting for diagnosed patients receiving IgG, annual estimated savings to the healthcare system is remarkable, at $87,888. Although assigning a dollar amount to a life is impossible, four agencies determined that amount to be $9.7 million, by considering certain factors that contribute to society including productivity, spending, healthcare costs, and taxes [31]. This estimate assumes a lifespan of 70 years on average with a value of $135,714 per year. These cost savings emphasize the critical need for early and accurate diagnosis and appropriate treatment, which ultimately lead to improved outcomes and lives saved.

National health spending in the US from 2019 to 2028 is expected to rise at a 5.4% average annual rate, reaching $6.2 trillion [32]. Individual spending rose at a 4.3% average annual rate between 2014 and 2018 [23]. Notably, costs were restrained by utilization of outpatient care and a growing proportion of the US population receiving coverage through the Affordable Care Act. Importantly, the economic impacts were determined to be even greater than our previous estimates in a study assessing the economic effect of infections in PI patients obtaining IVIG therapy [33]. Cost-effective strategies in all aspects of healthcare, from diagnosis to treatment, are imperative to reduce the economic burden on individuals and the healthcare system.

### JMF initiatives

SPIRIT^®^ (Software for Primary Immunodeficiency Recognition, Intervention, and Tracking) Analyzer was developed by JMF as a cost-effective strategy to further expedite the early diagnosis of patients with PI. The SPIRIT^®^ Analyzer identifies at-risk patients by matching 352 ICD-10 codes to JMF’s 10 Warning Signs of PI (Figs. 1, 2). The software analyzes over one million claims per hour in large existing datasets and establishes low-, medium-, and high-risk categories by calculating risk points. It also uses current National Drug Pharmacy and Healthcare Common Procedure codes and data on dosage and frequency to calculate antibiotic use risk scores. The software was recently used prospectively in a contained health care coverage system [34] and is made available to providers and healthcare insurance companies as a public service by JMF. The SPIRIT^®^ Analyzer provides an opportunity to notify providers of patients at risk for having a PI. These at-risk patients can then ideally obtain timely access to appropriate assessment, which ultimately leads to shortened time to diagnoses, improved outcomes and QOL. This early identification of patients with PI is predicted to be associated with considerable healthcare cost savings and the JMF is hopeful for increased utilization of this tool.

**Fig. 9:**
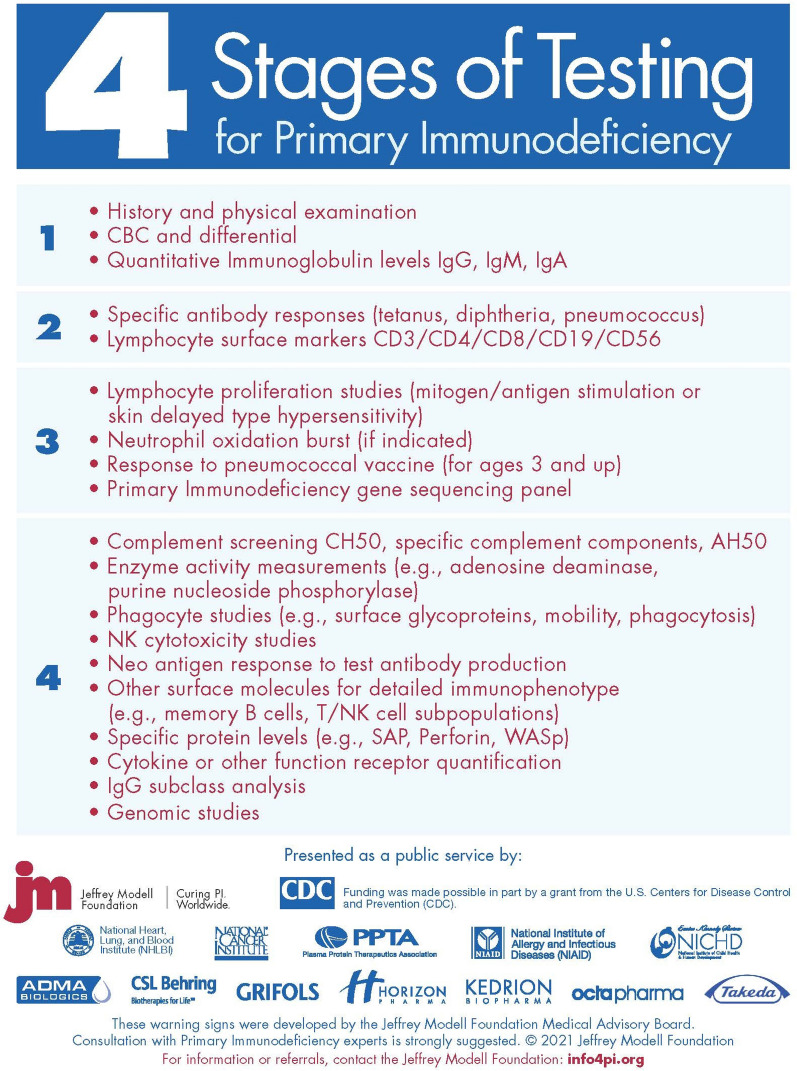
Physician Algorithm: JMF’s 4 stages of testing for PI

The initiation of SCID newborn screening programs, improvements in diagnostics, and advancements in genomic technologies over the past few decades have allowed for better prevalence estimates and have resulted in improved comprehension of PI and the causal mechanisms leading to monogenic defects of the immune system. To further improve diagnostics, and therefore our overall understanding of PI and its prevalence, JMF’s Physician Algorithm: 4 Stages of Testing for PI has recently been updated in 2021, as seen in Fig. 9. However, there are still many undiscovered PIs. More of these causative defects will be discovered through further investigation of gene candidates and with the advancement of NGS technologies, which will improve our comprehension of disease mechanisms and of the immune system overall [35]. It is vital that global access advances at equal pace with these genetic technologies, to limit diagnostic inequalities.

Suspected PI patients with no genetic diagnosis often undergo a costly, time-consuming, and arduous diagnostic odyssey, delaying proper disease management and treatment, prolonging suffering, and decreasing QOL. NGS, which can mitigate this diagnostic odyssey, is unfortunately frequently unobtainable due to cost and inaccessibility. In 2019, to address the issue of accessibility and contribute to the advancement of these technologies, JMF initiated a free NGS pilot program for JMCN patients clinically diagnosed with a PI [36], which aimed to identify a specific defect, providing medical professionals a precise diagnosis for appropriate management and treatment, and highlight the value of PI NGS through the JMCN. Twenty-one JMCN sites, in 10 countries, were invited to participate. One hundred and fifty-eight patients were tested, using a commercially available PI NGS panel, which included 207 genes at the time, and 21% received a molecular diagnosis. Through NGS, clinical diagnosis, disease management, treatment, and genetic counseling were altered in a substantial number of patients. Importantly, nearly half of the patients experienced a change in outcomes and there was an available therapy for nearly all diagnosed patients. This pilot [36] demonstrated the cost-efficiency, utility, and critical importance of NGS for PI.

Building on the success of the pilot program, JMF rolled this program out globally in early 2020, offering the entire JMCN an opportunity to participate. The gene panel was expanded from 207 to 407 genes, representing ~ 95% of the PI genes recognized by the IUIS. The program is ongoing, and critical feedback is being collected to further assess access challenges and impact on disease management and treatment. The program has been impactful thus far, and we are enthusiastic about its continuing success as we look to expand access beyond the JMCN globally. We believe the initiative showcases the impact, importance, and necessity of NGS for suspected PI patients, as well as the benefit of a true network of expert immunologists.

With the continued advancement of molecular technology, genome sequencing is becoming more common [37]. The resulting increasing numbers of medically actionable genotypes are driving the concept of “personalized medicine”. As this technology advances, rare genotypes will be identified and known genotypes will be “immunophenotypically” expanded [37]. Soon, the complete genomes of newborns could be routinely sequenced [38], offering unprecedented insight into and foresight for a variety of health conditions including those of the immune system.

At present, 450 PI defects have been identified [2, 17, 39]. Over the last 5 years, at least 100 new genes were discovered by investigators within the JMCN, through molecular diagnosis, genetic sequencing, and advanced immunobiological investigations. The JMCN and its member physicians and investigators have continued to advance toward novel cures, including innovations in re-programming SCID mutations in hematopoietic stem cells using CRISPR technology and genome editing [40], and Antiviral T cell immunotherapy [41, 42]. This comprehensive global analysis of physician-reported data on patients with PI demonstrates an increase in the diagnosis of numerous genotypes throughout the JMCN. In addition to providing the foundation for early diagnosis and appropriate treatments, the JMCN serves as a longstanding and growing platform for collaboration and cutting-edge research, with coordinated and open access to expert immunologists, to promote further meaningful advancements in the field of PI, including gene discovery [10, 14, 43, 44].

## Conclusions

The JMCN has steadily and substantially grown for more than two decades and continues to meaningfully influence the global Immunology community. In addition to building an extensive global patient database, the expanding JMCN serves as a unique and critical resource, providing the infrastructure for earliest diagnosis, optimized treatments, and implementation of standard-of-care and best practices. The JMCN provides a much-needed platform that facilitates the education of physicians and patients, awareness initiatives, and research advances, through collaboration and connectivity, ultimately resulting in improved outcomes and QOL for patients with PI.

## Supplementary Information

**Additional file 1: Figure S1.** 2020–2021 Global Survey on Primary Immunodeficiencies. A survey developed by JMF for members of the JMCN following the most recent Classification of PI from the IUIS Expert Committee.

**Additional file 2: Table S1.** Percentage of Missing Responses by Variable. The percentage of missing responses by variable, which ranges from 0.0 to 97.3% (n = 300). Variables correspond to the 2020–2021 Global Survey on Primary Immunodeficiencies. To note, for all variables other than demographics, a missing response was interpreted as a “zero” in that the physician did not have any patients having that particular condition or receiving that particular treatment.

## Data Availability

Not applicable; all data used for this analysis are included in this published article.

## Abbreviations

PI: Primary immunodeficiencies
IEI: Inborn error of immunity
SCID: Severe combined immunodeficiency
QOL: Quality of life
JMF: Jeffrey Modell Foundation
JMCN: Jeffrey Modell Centers Network
IUIS: International Union of Immunological Societies
NGS: Next generation sequencing
OMIM: Online Mendelian inheritance in man
HSCT: Hematopoietic stem cell transplantation
HIPAA: Health Insurance Portability and Accountability Act
ER: Emergency room
AHRQ: Agency for healthcare research and quality
MEPS: Medical Expenditure Panel Survey
AHA: American Hospital Association
HCCI: Health Care Cost Institute
HCUP: Hospital Cost and Utilization Project
US: United States
CVID: Common variable immunodeficiency
IgG: Immunoglobulin therapy
SCIG: Subcutaneous immunoglobulin therapy
IVIG: Intravenous immunoglobulin therapy
PEG-ADA: Polyethylene glycol-conjugated adenosine deaminase
MRD: Matched related donor
MUD: Matched unrelated donor
mMUD: Mismatched unrelated donor
SPIRIT: Software for primary immunodeficiency recognition, intervention, and tracking
ICD: International classification of diseases
CRISPR: Clustered regularly interspaced short palindromic repeats
