# Supplementary material for: Growth in diagnosis and treatment of primary immunodeficiency within the global Jeffrey Modell Centers Network
Source: Allergy Asthma Clin Immunol. 2022 Mar 4;18:19. doi: 10.1186/s13223-022-00662-6 (PMC8896271; doi:10.1186/s13223-022-00662-6)
Supplement: Supplementary file 1 — Additional file 1: Figure S1. 2020–2021 Global Survey on Primary Immunodeficiencies. A survey developed by JMF for members of the JMCN following the most recent Classification of PI from the IUIS Expert Committee. [file 13223_2022_662_MOESM1_ESM.pdf]

2020-2021 Global Survey on Primary Immunodeficiencies

Jeffrey Modell Centers Network

City, State, Country: ______________________________

Name of Institution: ______________________________

Name of Physician(s) (Last, First): ______________________________

1. Total number of patients being followed	
2. Total number of patients identified with a specific PI defect	
3. Total number of patients receiving IgG:	
A) IVIG - Clinic	
B) IVIG - Home	
C) SCIG	
D) Other	
4. Total number of patients treated by Gene Therapy	
5. Total number of patients treated with PEG-ADA	

6. Total number of patients treated by Transplant	
Donor Type:	
A) MRD	
B) MUD	
C) mMUD	
D) Parental Haplo	
Stem Cell Source:	
A) BM	
B) PBSC	
C) Cord	
D) Other (please specify)	

For Tables I - X, please enter the number of patients followed in the box to the right of the specified gene. Available OMIM numbers are provided and linked within each table.

TABLE I. IMMUNODEFICIENCIES AFFECTING CELLULAR AND HUMORAL IMMUNITY

1.	ADA (Adenosine deaminase deficiency), AR	OMIM 608958	1.
2.	AK2 (AK2 Defect), AR	OMIM 103020	2.
3.	B2M (MHC class I deficiency), AR	OMIM 109700	3.
4.	BCL10 (BCL10 deficiency), AR	OMIM 616098	4.
5.	CARD 11 (CARD11 deficiency), AR LOF	OMIM 615206	5.
6.	CD3D (CD3d deficiency), AR	OMIM 186790	6.
7.	CD3E (CD3ε deficiency), AR	OMIM 186830	7.
8.	CD3G (CD3γ deficiency), AR	OMIM 186740	8.
9.	CD3Z (CD3ζ deficiency), AR	OMIM 186780	9.
10.	CD40 (CD40 deficiency), AR	OMIM 606843	10.
11.	CD40LG (CD40 ligand (CD154) deficiency), XL	OMIM 308230	11.
12.	CD8A (CD8 deficiency), AR	OMIM 186910	12.
13.	CIITA (MHC class II deficiency group A, B, C, D), AR	OMIM 600005	13.
14.	CORO1A (Coronin-1A deficiency), AR	OMIM 605000	14.
15.	DCLRE1C (Artemis deficiency), AR	OMIM 605988	15.
16.	DOCK2 (DOCK2 deficiency), AR	OMIM 603122	16.
17.	DOCK8 (DOCK8 deficiency), AR	OMIM 243700	17.
18.	FCHO1 (FCHO1 deficiency), AR	OMIM 613437	18.
19.	ICOS (ICOS deficiency), AR	OMIM 604558	19.
20.	ICOSLG (ICOSL deficiency), AR	OMIM 605717	20.
21.	IKBKB (IKBKB deficiency), AR	OMIM 615592	21.
22.	IKZF1 (IKAROS deficiency), AD DN	OMIM 603023	22.
23.	IL21 (IL-21 deficiency), AR	OMIM 615767	23.
24.	IL21R (IL-21R deficiency), AR	OMIM 615207	24.
25.	IL2RG (γc Deficiency, γc SCID, CD132 deficiency), XL	OMIM 308380	25.
26.	IL7R (IL7Rα deficiency), AR	OMIM 146661	26.
27.	ITK (ITK deficiency), AR	OMIM 186973	27.
28.	JAK3 (JAK3 deficiency), AR	OMIM 600173	28.
29.	LAT (LAT deficiency), AR	OMIM 602354	29.
30.	LCK (LCK deficiency), AR	OMIM 615758	30.
31.	LIG4 (DNA ligase IV deficiency), AR	OMIM 601837	31.
32.	MALT1 (MALT1 deficiency), AR	OMIM 615468	32.
33.	MAP3K14 (NIK deficiency), AR	OMIM 604655	33.
34.	MSN (Moesin deficiency), XL	OMIM 300988	34.
35.	NHEJ1 (Cernunnos/XLF deficiency), AR	OMIM 611290	35.
36.	POLD 1 (Polymerase δ deficiency), AR	OMIM 174761	36.
37.	POLD 2 (Polymerase δ deficiency), AR	OMIM 600815	37.
38.	PRKDC (DNA PKcs deficiency), AR	OMIM 615966	38.
39.	PTPRC (CD45 Deficiency), AR	OMIM 151460	39.
40.	RAC2 (Activated RAC2 defect), AD GOF	OMIM 602049	40.
41.	RAG1 (RAG deficiency), AR	OMIM 179615	41.
42.	RAG2 (RAG deficiency), AR	OMIM 179616	42.
43.	REL (c-Rel deficiency), AR	OMIM 164910	43.
44.	RELA (RelA haploinsufficiency), AD	OMIM 618287	44.
45.	RELB (RelB deficiency), AR	OMIM 604758	45.
46.	RFX5 (MHC class II deficiency group A, B, C, D), AR	OMIM 601863	46.
47.	RFXANK (MHC class II deficiency group A, B, C, D), AR	OMIM 603200	47.
48.	RFXAP (MHC class II deficiency group A, B, C, D), AR	OMIM 601861	48.
49.	RHOH (RHOH deficiency), AR	OMIM 602037	49.
50.	STK4 (MST1 deficiency), AR	OMIM 614868	50.
51.	TAP1 (MHC class I deficiency), AR	OMIM 170260	51.
52.	TAP2 (MHC class I deficiency), AR	OMIM 170261	52.
53.	TAPBP (MHC class I deficiency), AR	OMIM 601962	53.
54.	TFRC (TFRC deficiency), AR	OMIM 616740	54.
55.	TNFRSF4 (OX40 deficiency), AR	OMIM 615593	55.
56.	TRAC (TCRα deficiency), AR	OMIM 615387	56.
57.	ZAP70 (ZAP-70 combined mutations), AR (LOF/GOF)	OMIM 617006	57.
58.	ZAP70 (ZAP-70 deficiency (ZAP70 LOF)), AR	OMIM 269840	58.
59.	Other Immunodeficiencies Affecting Cellular and Humoral Immunity: *Please also list in "Unspecified" section on Page 5		59.

TABLE II. COMBINED IMMUNODEFICIENCIES WITH ASSOCIATED OR SYNDROMIC FEATURES

1. 11q23del (Chromosome 11q deletion syndrome(Jacobsen syndrome)), AD	OMIM 147791	1.		37. NSMCE3 (NSMCE3 deficiency), AR	OMIM 608243	37.	
2. ARPC1B (Arp2/3-mediated filament branching defect), AR	OMIM 604223	2.		38. ORAI1 (ORAI-I deficiency), AR	OMIM 610277	38.	
3. ATM (Ataxia-telangiectasia), AR	OMIM 607585	3.		39. PGM3 (PGM3 Deficiency), AR	OMIM 172100	39.	
4. BCL11B (BCL11B deficiency), AD	OMIM 617237	4.		40. PMS2 (PMS2 Deficiency), AR	OMIM 600259	40.	
5. BLM (Bloom Syndrome), AR	OMIM 604610	5.		41. PNP (PNP deficiency), AR	OMIM 164050	41.	
6. CARD11 (CARD11 deficiency (heterozygous)), AD LOF	OMIM 617638	6.		42. POLE1 (FILS Syndrome), AR	OMIM 174762	42.	
7. CCBE1 (Hennekam-lymphangiectasia-lymphedema Syndrome), AR	OMIM 612753	7.		43. POLE2 (POLE2 deficiency), AR	OMIM 602670	43.	
8. CDCA7 (ICF3), AR	OMIM 609937	8.		44. RBCK1 (HOIL1 deficiency), AR	OMIM 610924	44.	
9. CHARGE Syndrome, unknown		9.		45. RMRP (Cartilage Hair Hypoplasia), AR	OMIM 157660	45.	
10. CHD7 (CHARGE Syndrome), AD	OMIM 608892	10.		46. RNF168 (RIDDLE Syndrome), AR	OMIM 612688	46.	
11. Del10p13-p14 (Chromosome 10p13-p14 deletion syndrome), AD	OMIM 601362	11.		47. RNF31 (HOIP deficiency), AR	OMIM 612487	47.	
12. DiGeorge Syndrome, unknown		12.		48. RNU4ATAC (MOPD1 deficiency (Roifman syndrome)), AR	OMIM 601428	48.	
13. DNMT3B (ICF1), AR	OMIM 602900	13.		49. SEMA3E (CHARGE Syndrome), AD	OMIM 608166	49.	
14. EPG5 (EPG5 deficiency (Vici syndrome)), AR	OMIM 615068	14.		50. SKIV2L (Tricho-Hepato-Enteric Syndrome (THES)), AR	OMIM 614602	50.	
15. ERBB2IP (ERBIN deficiency), AD	OMIM 606944	15.		51. SLC46A1 (SLC46A1/PCFT deficiency), AR	OMIM 229050	51.	
16. ERCC6L2 (Hebo deficiency), AR	OMIM 615667	16.		52. SMARCAL1 (Schimke Immuno-osseous dysplasia), AR	OMIM 606622	52.	
17. EXTL3 (EXTL3 deficiency), AR	OMIM 617425	17.		53. SP110 (VODI syndrome), AR	OMIM 604457	53.	
18. FAT4 (Hennekam-lymphangiectasia-lymphedema Syndrome), AR	OMIM 612411	18.		54. SPINK5 (Comel-Netherton syndrome), AR	OMIM 605010	54.	
19. FOXN1 (FOXN1 haploinsufficiency), AD	OMIM 600838	19.		55. STAT3 (AD-HIES STAT3 deficiency (Job syndrome)), AD LOF	OMIM 147060	55.	
20. FOXN1 (Winged helix nude FOXN1 deficiency), AR	OMIM 601705	20.		56. STAT5b (STAT5b deficiency), AD	OMIM 604260	56.	
21. GINS1 (GINS1 deficiency), AR	OMIM 610608	21.		57. STAT5b (STAT5b deficiency), AR	OMIM 245590	57.	
22. HELLS (ICF4), AR	OMIM 603946	22.		58. STIM1 (STIM-1 deficiency), AR	OMIM 605921	58.	
23. IKBKB (EDA-ID due to GOF mutation), AD GOF	OMIM 618204	23.		59. TBX1 (DiGeorge, Chromosome 22q11.2 deletion syndrome), AD	OMIM 602054	59.	
24. IKBKG (EDA-ID due to NEMO/IKBKG deficiency), XL	OMIM 300248	24.		60. TBX1 (TBX1 Deficiency), AD	OMIM 602054	60.	
25. IL6R (IL6 receptor deficieny), AR	OMIM 147880	25.		61. TCN2 (Transcobalamin 2 deficiency), AR	OMIM 613441	61.	
26. IL6ST (IL6 signal transducer (IL6ST) deficiency), AR	OMIM 618523	26.		62. TGFBR1 (Loeys-Dietz syndrome (TGFBR deficiency)), AD	OMIM 609192	62.	
27. KDM6A (Kabuki syndrome), XL	OMIM 300128	27.		63. TGFBR2 (Loeys-Dietz syndrome (TGFBR deficiency)), AD	OMIM 610168	63.	
28. KMT2A (KMT2A deficiency (Wiedemann-Steiner syndrome)), AD	OMIM 605130	28.		64. TTC37 (Tricho-Hepato-Enteric Syndrome (THES)), AR	OMIM 222470	64.	
29. KMT2D (Kabuki syndrome), AD	OMIM 602113	29.		65. TTC7A (Immunodeficiency with multiple intestinal atresias), AR	OMIM 609332	65.	
30. LIG1 (Ligase I deficiency), AR	OMIM 126391	30.		66. WAS (Wiskott-Aldrich syndrome (WAS LOF)), XL	OMIM 300392	66.	
31. MCM4 (MCM4 Deficiency), AR	OMIM 602638	31.		67. WIPF1 (WIP Deficiency), AR	OMIM 602357	67.	
32. MTHFD1 (MTHFD1 deficiency), AR	OMIM 172460	32.		68. ZBTB24 (ICF2), AR	OMIM 614064	68.	
33. MYSM1 (MYSM1 deficiency), AR	OMIM 612176	33.		69. ZNF341 (ZNF341 deficiency AR-HIES), AR	OMIM 618282	69.	
34. NBS1 (Nijmegen breakage syndrome), AR	OMIM 602667	34.		70. Other Combined Immunodeficiencies with syndromic features:		70.	
35. NFE2L2 (Activating de novo mutations in nuclear factor, erythroid 2-like)	OMIM 617744	35.		*Please also list in "Unspecified" section on Page 5			
36. NFKBIA (EDA-ID due to IKBA GOF mutation), AD GOF	OMIM 164008	36.					

TABLE III. PREDOMINANTLY ANTIBODY DEFICIENCIES

1. AICDA (AID deficiency), AR	OMIM 605258	1.		26. ATP6AP1 (ATP6AP1 deficiency), XL	OMIM 300972	26.	
2. TNFRSF13C (BAFF receptor deficiency), AR	OMIM 606269	2.		27. TRNT1 (TRNT1 deficiency), AR	OMIM 612907	27.	
3. BLNK (BLNK deficiency), AR	OMIM 604515	3.		28. TNFS12 (TWEAK deficiency), AD	OMIM 602695	28.	
4. BTK (BTK deficiency, XLA) XL	OMIM 300300	4.		29. UNG (UNG deficiency), AR	OMIM 191525	29.	
5. CARD11 (CARD11 GOF), AD GOF	OMIM 616452	5.		30. IGLL1 (λ5 deficiency), AR	OMIM 146770	30.	
6. CD19 (CD19 deficiency), AR	OMIM 107265	6.		31. PIK3CD (p110δ deficiency), AR	OMIM 602839	31.	
7. CD20 (CD20 deficiency), AR	OMIM 112210	7.		32. TCF3 (E47 transcription factor deficiency), AD	OMIM 616941	32.	
8. CD21 (CD21 deficiency), AR	OMIM 120650	8.		33. TCF3 (E47 transcription factor deficiency), AR	OMIM 147141	33.	
9. CD81 (CD81 deficiency), AR	OMIM 186845	9.		34. SLC39A7 (ZIP7 deficiency), AR	OMIM 601416	34.	
10. Transient hypogammaglobulinemia of Infancy		10.		35. TOP2B (Hoffman syndrome/TOP2B deficiency), AD	OMIM 126431	35.	
11. CD79A (Igα deficiency), AR	OMIM 112205	11.		36. CVID, unknown		36.	
12. CD79B (Igβ deficiency), AR	OMIM 147245	12.		37. PTEN (PTEN deficiency (LOF)), AD	OMIM 158350	37.	
13. IgG subclass deficiency with IgA deficiency		13.		38. TNFRSF13B (TACI deficiency), AR or AD	OMIM 604907	38.	
14. Isolated IgG subclass deficiency		14.		39. NFKB2 (NFKB2 deficiency), AD	OMIM 615577	39.	
15. Ig heavy chain mutations and deletions, AR		15.		40. IKZF1 (IKAROS deficiency), AD	OMIM 603023	40.	
16. IGKC (kappa chain deficiency), AR	OMIM 147200	16.		41. IRF2BP2 (IRF2BP2 deficiency), AD	OMIM 615332	41.	
17. INO80 (INO80 deficiency), AR	OMIM 610169	17.		42. ARHGEF1 (ARHGEF1 deficiency), AR	OMIM 618459	42.	
18. IGHM (μheavy chain deficiency), AR	OMIM 147020	18.		43. SH3KBP1 (SH3KBP1 (CIN85) deficiency), XL	OMIM 300310	43.	
19. MOGS (MOGS deficiency), AR	OMIM 601336	19.		44. SEC61A1 (SEC61A1 deficiency), AD	OMIM 609213	44.	
20. MSH6 (MSH6 deficiency), AR	OMIM 600687	20.		45. RAC2 (RAC2 deficiency), AR	OMIM 602049	45.	
21. NFKB1 (NFKB1 deficiency), AD	OMIM 164011	21.		46. AICDA (AID deficiency), AD	OMIM 605257	46.	
22. PIK3R1 (Activated p110δ syndrome (APDS2)), AD	OMIM 616005	22.		47. Selective IgA deficiency		47.	
23. PIK3R1 (p85 deficiency), AR	OMIM 615214	23.		48. Selective IgM deficiency		48.	
24. PIK3CD GOF (Activated p110δ syndrome (APDS1)), AD	OMIM 615513	24.		49. Other Predominantly Antibody Deficiencies:		49.	
25. Specific antibody deficiency (normal Ig and B cells)		25.		*Please also list in "Unspecified" section on Page 5			

TABLE IV. DISEASES OF IMMUNE DYSREGULATION

1. AIRE (APECED (APS-1), AR or AD	OMIM 240300	1.		24. LRBA (LRBA deficiency), AR	OMIM 606453	24.	
2. AP3B1 (Hermansky-Pudlak syndrome type 2), AR	OMIM 603401	2.		25. LYST (Chediak-Higashi syndrome), AR	OMIM 606897	25.	
3. AP3D1 (Hermansky-Pudlak syndrome type 10), AR	OMIM 617050	3.		26. MAGT1 (XMEN), XL	OMIM 300853	26.	
4. BACH2 (BACH2 deficiency), AD	OMIM 605394	4.		27. NFAT5 (NFAT5 haploinsufficiency), AD	OMIM 604708	27.	
5. CARMIL2 (RLTPR deficiency), AR	OMIM 610859	5.		28. PEPD (Prolidase deficiency), AR	OMIM 613230	28.	
6. CASP10 (ALPS-Caspase 10), AD	OMIM 601762	6.		29. PRF1 (Perforin deficiency (FHL2)), AR	OMIM 170280	29.	
7. CASP8 (ALPS-Caspase 8), AR	OMIM 601763	7.		30. PRKCD (PRKCD deficiency), AR	OMIM 615559	30.	
8. CD27 (CD27 deficiency), AR	OMIM 615122	8.		31. RAB27A (Griscelli syndrome type 2), AR	OMIM 603868	31.	
9. CD70 (CD70 deficiency), AR	OMIM 602840	9.		32. RASGRP1 (RASDRP1 deficiency), AR	OMIM 603962	32.	
10. CTLA4 (CTLA4 haploinsufficiency (ALPS-V)), AD	OMIM 123890	10.		33. RIPK1 (RIPK1), AR	OMIM 618108	33.	
11. CTPS1 (CTPS1 deficiency), AR	OMIM 615897	11.		34. SH2D1A (SAP deficiency (XLP1), XL	OMIM 300490	34.	
12. DEF6 (DEF6 deficiency), AR	OMIM 610094	12.		35. SLC7A7 (SLC7A7 deficiency), AR	OMIM 222700	35.	
13. FAAP24 (FAAP24 deficiency), AR	OMIM 610884	13.		36. STAT3 (STAT3 GOF mutation), AD GOF	OMIM 102582	36.	
14. FADD (FADD deficiency), AR	OMIM 602457	14.		37. STX11 (Syntaxin 11 deficieny (FHL4)), AR	OMIM 605014	37.	
15. FERMT1 (FERMT1 deficiency), AR	OMIM 173650	15.		38. STXBP2 (STXBP2/Munc18-2 deficiency (FHL5)), AR or AD	OMIM 601717	38.	
16. FOXP3 (IPEX syndrome), XL	OMIM 300292	16.		39. TGFB1 (TGFB1 deficiency), AR	OMIM 618213	39.	
17. IL10 (IL-10 deficiency), AR	OMIM 124092	17.		40. TNFRSF6 (ALPS-FAS), AD or AR	OMIM 134637	40.	
18. IL10RA (IL-10R deficiency), AR	OMIM 146933	18.		41. TNFRSF9 (CD137 deficiency (41BB)), AR	OMIM 602250	41.	
19. IL10RB (IL-10R deficiency), AR	OMIM 123889	19.		42. TNFSF6 (ALPS-FASLG), AR	OMIM 134638	42.	
20. IL2RA (CD25 deficiency), AR	OMIM 147730	20.		43. TPP2 (Tripeptidyl-peptidase II deficiency), AR	OMIM 190470	43.	
21. IL2RB (CD122 deficiency), AR	OMIM 618495	21.		44. UNC13D (UNC13D/Munc12-4 deficiency (FHL3)), AR	OMIM 608897	44.	
22. ITCH (ITCH deficiency), AR	OMIM 606409	22.		45. XIAP (XIAP deficiency (XLP2), XL	OMIM 300079	45.	
23. JAK1 (JAK1 GOF), AD GOF	OMIM 147795	23.		46. Other Diseases of Immune Dysregulation:		46.	
				*Please also list in "Unspecified" section on Page 5			

TABLE V. CONGENITAL DEFECTS OF PHAGOCYTE NUMBER OR FUNCTION

1.	ACTB (b-Actin deficiency), AD	OMIM 102630	1.		23.	HYOU1 (HYOU1 deficiency), AR	OMIM 601746	23.	
2.	CEBPE (Specific granule deficiency), AR	OMIM 189965	2.		24.	ITGB2 (Leukocyte adhesion deficiency type 1 (LAD1)), AR	OMIM 600065	24.	
3.	CFTR (Cystic fibrosis), AR	OMIM 602421	3.		25.	JAGN1 (JAGN1 deficiency), AR	OMIM 616012	25.	
4.	CLBP (3-Methylglutaconic aciduria), AR	OMIM 616254	4.		26.	LAMTOR2 (P14/LAMTOR2 deficiency), AR	OMIM 610389	26.	
5.	CSF2RA (Pulmonary alveolar proteinosis), XL	OMIM 300770	5.		27.	MKL1 (Neutropenia with combined immune deficiency), AR	OMIM 606078	27.	
6.	CSF2RB (Pulmonary alveolar proteinosis), AR	OMIM 614370	6.		28.	NCF1 (CGD), AR	OMIM 608512	28.	
7.	CSF3R (G-CSF receptor deficiency), AR	OMIM 138971	7.		29.	NCF2 (CGD), AR	OMIM 608515	29.	
8.	CTSC (Papillon-Lefèvre syndrome), AR	OMIM 602365	8.		30.	NCF4 (CGD), AR	OMIM 613960	30.	
9.	CYBA (CGD), AR	OMIM 608508	9.		31.	RAC2 (Rac2 deficiency), AD LOF	OMIM 608203	31.	
10.	CYBB (X-linked CGD (gp91 phox)), XL	OMIM 306400	10.		32.	SBDS (Shwachman-Diamond Syndrome), AR	OMIM 607444	32.	
11.	CYBC1 (CGD), AR	OMIM 618334	11.		33.	SLC35C1 (Leukocyte adhesion deficiency type 2 (LAD2)), AR	OMIM 605881	33.	
12.	DNAJC21 (Shwachman-Diamond Syndrome), AR	OMIM 617052	12.		34.	SMARCD2 (SMARCD2 deficiency), AR	OMIM 601736	34.	
13.	EFL1 (Shwachman-Diamond Syndrome), AR	OMIM 617941	13.		35.	SRP54 (SRP54 deficiency), AD	OMIM 604857	35.	
14.	ELANE (Elastase deficiency (Severe congenital neutropenia [SCN] 1)), AD	OMIM 130130	14.		36.	TAZ (Barth syndrome), XL	OMIM 300394	36.	
15.	FERMT3 (Leukocyte adhesion deficiency type 3 (LAD3)), AR	OMIM 607901	15.		37.	USB1 (Clericuzio syndrome), AR	OMIM 613276	37.	
16.	FPR1 (Localized juvenile periodontitis), AR	OMIM 136537	16.		38.	VPS13B (Cohen syndrome), AR	OMIM 607817	38.	
17.	G6PC3 (G6PC3 deficiency (SCN4)), AR	OMIM 611045	17.		39.	VPS45 (VPS45 deficiency (SCN5)), AR	OMIM 610035	39.	
18.	G6PD (G6PD deficiency class I), XL	OMIM 305900	18.		40.	WAS (X-linked neutropenia/myelodysplasia), XL GOF	OMIM 300299	40.	
19.	G6PT1 (Glycogen storage disease type 1b), AR	OMIM 602671	19.		41.	WDR1 (WDR1 deficiency), AR	OMIM 604734	41.	
20.	GATA2 (GATA2 deficiency), AD	OMIM 137295	20.		42.	Other Congenital Defects of Phagocytes:		42.	
21.	GFI1 (GFI1 deficiency (SCN2)), AD	OMIM 600871	21.			*Please also list in "Unspecified" section on Page 5			
22.	HAX1 (HAX1 deficiency (Kostmann Disease (SCN3))), AR	OMIM 605998	22.						

TABLE VI. DEFECTS IN INTRINSIC AND INNATE IMMUNITY

1.	APOL1 (Trypanosomiasis), AD	OMIM 603743	1.		35.	NBAS (Acute liver failure due to NBAS deficiency), AR	OMIM 608025	35.	
2.	CARD9 (CARD9 deficiency), AR	OMIM 607212	2.		36.	NCSTN (Hidradenitis suppurativa), AD	OMIM 605254	36.	
3.	CIB1 (CIB1 deficiency (HPV))	OMIM 618267	3.		37.	OSTM1 (Osteopetrosis), AR	OMIM 607649	37.	
4.	CLCN7 (Osteopetrosis), AR	OMIM 602727	4.		38.	PLEKHM1 (Osteopetrosis), AR	OMIM 611466	38.	
5.	CXCR4 (WHIM Syndrome (HPV)), AD GOF	OMIM 162643	5.		39.	POLR3A (RNA polymerase III deficiency), AD	OMIM 614258	39.	
6.	CYBB (Macrophage gp91 phox deficiency (MSMD)), XL	OMIM 300645	6.		40.	POLR3C (RNA polymerase III deficiency), AD	OMIM 617454	40.	
7.	DBR1 (DBR1 deficiency (HSE)), AR	OMIM 607024	7.		41.	POLR3F (RNA polumerase III deficiency), AD	OMIM 617455	41.	
8.	FCGR3A (CD16 deficiency), AR	OMIM 146740	8.		42.	PSEN (Hidradenitis suppurativa), AD	OMIM 613737	42.	
9.	HMOX (Isolated congenital asplenia (ICA)), AR	OMIM 141250	9.		43.	PSENEN (Hidradenitis suppurativa), AD	OMIM 613736	43.	
10.	IFIH1 (MDA5 deficiency), AR LOF	OMIM 606951	10.		44.	RANBP2 (Acute necrotizing encephalopathy), AR	OMIM 601181	44.	
11.	IFNAR1 (IFNAR1 deficiency), AR	OMIM 107450	11.		45.	RORC (RORγt deficiency (MSMD)), AR	OMIM 602943	45.	
12.	IFNAR2 (IFNAR2 deficiency), AR	OMIM 602376	12.		46.	RPSA (Isolated congenital asplenia (ICA)), AD	OMIM 271400	46.	
13.	IFNGR1 (IFN-γ receptor 1 deficiency (MSMD)), AD	OMIM 615978	13.		47.	SNX10 (Osteopetrosis), AR	OMIM 614780	47.	
14.	IFNGR1 (IFN-γ receptor 1 deficiency (MSMD)), AR	OMIM 209950	14.		48.	SPPL2A (SPPL2a deficiency (MSMD)), AR	OMIM 608238	48.	
15.	IFNGR2 (IFN-γ receptor 2 deficiency (MSMD)), AR	OMIM 147569	15.		49.	STAT1 (STAT1 deficiency (MSMD)), AD LOF	OMIM 614892	49.	
16.	IL12B (IL-12p40 (IL-12 and IL-23) deficiency (MSMD)), AR	OMIM 161561	16.		50.	STAT1 (STAT1 deficiency), AR LOF	OMIM 600555	50.	
17.	IL12RB1 (IL-12 and IL-23 receptor β1 chain deficiency (MSMD)), AR	OMIM 601604	17.		51.	STAT1 (STAT1 GOF (CMC)), AD GOF	OMIM 600555	51.	
18.	IL12RB2 (IL-12Rβ2 deficiency (MSMD)), AR	OMIM 601642	18.		52.	STAT2 (STAT2 deficiency), AR	OMIM 600556	52.	
19.	IL17F (IL-17F deficiency (CMC)), AD	OMIM 606496	19.		53.	TBK1 (TBK1 deficiency (HSE)), AD	OMIM 604834	53.	
20.	IL17RA (IL-17RA deficiency (CMC)), AR	OMIM 605461	20.		54.	TCIRG1 (Osteopetrosis), AR	OMIM 604592	54.	
21.	IL17RC (IL-17RC deficiency (CMC)), AR	OMIM 610925	21.		55.	TICAM1 (TRIF deficiency (HSE)), AD or AR	OMIM 607601	55.	
22.	IL18BP (IL-18BP deficiency), AR	OMIM 604113	22.		56.	TIRAP (TIRAP deficiency), AR	OMIM 614382	56.	
23.	IL23R (IL-23R deficiency (MSMD)), AR	OMIM 607562	23.		57.	TLR3 (TLR3 deficiency (HSE)), AD or AR	OMIM 613002	57.	
24.	IRAK1 (IRAK1 deficiency), XL	OMIM 300283	24.		58.	TMC6 (EVER1 deficiency (HPV)), AR	OMIM 605828	58.	
25.	IRAK4 (IRAK4 deficiency), AR	OMIM 606883	25.		59.	TMC8 (EVER2 deficiency (HPV))	OMIM 605829	59.	
26.	IRF3 (IRF3 deficiency (HSE)), AD	OMIM 616532	26.		60.	TNFRSF11A (Osteopetrosis), AR	OMIM 603499	60.	
27.	IRF4 (IRF4 haploinsufficiency), AD	OMIM 601900	27.		61.	TNFSF11 (Osteopetrosis), AR	OMIM 602642	61.	
28.	IRF7 (IRF7 deficiency), AR	OMIM 605047	28.		62.	TRAF3 (TRAF3 deficiency (HSE)), AD	OMIM 601896	62.	
29.	IRF8 (IRF8 defiency (MSMD)), AD	OMIM 614893	29.		63.	TRAF3IP2 (ACT1 deficiency), AR	OMIM 607043	63.	
30.	IRF8 (IRF8 defiency (MSMD)), AR	OMIM 226990	30.		64.	TYK2 (P1104A TYK2 homozygosity (MSMD)), AR	OMIM 176941	64.	
31.	IRF9 (IRF9 deficiency), AR	OMIM 147574	31.		65.	TYK2 (Tyk2 deficiency (MSMD)), AR	OMIM 611521	65.	
32.	ISG15 (ISG15 deficiency (MSMD)), AR	OMIM 147571	32.		66.	UNC93B1 (UNC93B1 deficiency (HSE)), AR	OMIM 608204	66.	
33.	JAK1 (JAK1 deficiency (MSMD)), AR LOF	OMIM 147795	33.		67.	NK Cell Deficiency		67.	
34.	MYD88 (MyD88 deficiency), AR	OMIM 602170	34.		68.	Other Defects in Innate Immunity:		68.	
						*Please also list in "Unspecified" section on Page 5			

TABLE VII. AUTOINFLAMMATORY DISORDERS

1. ADAM17 (ADAM17 deficiency), AR	OMIM 614328	1.		25. SAMHD1 (SAMHD1 deficiency (AGS5)), AR	OMIM 606754	25.	
2. IFIH1 (AGS7), AD GOF	OMIM 615846	2.		26. USP18 (USP18 deficiency), AR	OMIM 607057	26.	
3. SH3BP2 (Cherubism), AD	OMIM 118400	3.		27. NLRP3 (NOMID or CINCA), AD GOF	OMIM 607115	27.	
4. NLRP3 (Familial cold autoinflammatory syndrome 1), AD GOF	OMIM 120100	4.		28. ADA2 (ADA2 deficiency), AR	OMIM 607575	28.	
5. DNASE2 (DNAse II deficiency), AR	OMIM 126350	5.		29. LPIN2 (Majeed syndrome), AR	OMIM 609628	29.	
6. MEFV (Familial Mediterranean fever), AD	OMIM 134610	6.		30. PSMG2 (CANDLE), AR	OMIM 609702	30.	
7. TNFRSF1A (TNF receptor-associated periodic syndrome (TRAPS)), AD	OMIM 142680	7.		31. RNASEH2B (RNASEH2B deficiency (AGS2)), AR	OMIM 610326	31.	
8. ADAR1 (ADAR1 deficiency, AGS6)), AR	OMIM 146920	8.		32. RNASEH2C (RNASEH2C deficiency (AGS3)), AR	OMIM 610330	32.	
9. OAS1 (OAS1 deficiency), AD GOF	OMIM 164350	9.		33. NLRP12 (Familial cold autoinflammatory syndrome 2), AD GOF	OMIM 611762	33.	
10. ACP5 (SPENCD), AR	OMIM 171640	10.		34. TMEM173 (SAVI), AR	OMIM 612374	34.	
11. ALP1 (ALP1 deficiency), AR	OMIM 171740	11.		35. IL1RN (DIRA), AR	OMIM 612852	35.	
12. NOD2 (Blau syndrome), AD	OMIM 186580	12.		36. IL36RN (DITRA), AR	OMIM 614204	36.	
13. NLRP3 (Muckle-Wells syndrome), AD GOF	OMIM 191900	13.		37. DNASE1L3 (DNASE1L3 deficiency), AR	OMIM 614420	37.	
14. MEFV (Familial Mediterranean fever), AR LOF	OMIM 249100	14.		38. PLCG2 (FCAS3, or APLAID), AD GOF	OMIM 614468	38.	
15. PSMB8 (CANDLE), AR and AD	OMIM 256040	15.		39. PLCG2 (PLAID), AD GOF	OMIM 614878	39.	
16. MVK (Mevalonate kinase deficiency (Hyper IgD syndrome), AR	OMIM 260920	16.		40. NLRP1 (NLRP1 GOF), AD GOF	OMIM 615225	40.	
17. POLA1 (X-linked reticulate pigmentary disorder), XL	OMIM 301220	17.		41. OTULIN (Otulipenia/ORAS), AR	OMIM 615712	41.	
18. COPA (COPA defect), AD	OMIM 601924	18.		42. AP1S3 (AP1S3 deficiency), AR	OMIM 615781	42.	
19. CARD14 (CAMPS (CARD14 mediated psoriasis)), AD	OMIM 602723	19.		43. NLRC4 (NLRC4-MAS), AD GOF	OMIM 616050	43.	
20. SLC29A3 (SLC29A3 mutation), AR	OMIM 602782	20.		44. NLRC4 (Familial cold autoinflammatory syndrome 4), AD GOF	OMIM 616115	44.	
21. PSTPIP1 (PAPA syndrome, hyperzincemia, & hypercalprotectinemia), AD	OMIM 604416	21.		45. TNFAIP (A20 deficiency), AD	OMIM 616744	45.	
22. RNASEH2A (RNASEH2A deficiency (AGS4)), AR	OMIM 606034	22.		46. NLRP1 (NLRP1 deficiency), AR	OMIM 617388	46.	
23. TRIM22 (TRIM22), AR	OMIM 606559	23.		47. HAVCR2 (TIM3 deficiency), AR	OMIM 618398	47.	
24. TREX1 (TREX1 deficiency (AGS1)), AR	OMIM 606609	24.		48. Other Autoinflammatory Disorders: *Please also list in "Unspecified" section on Page 5		48.	

TABLE VIII. COMPLEMENT DEFICIENCIES

1. C1QA (C1q deficiency), AR	OMIM 120550	1.		20. CD55 (CD55 deficiency (CHAPEL disease)), AR	OMIM 125240	20.	
2. C1QB (C1q deficiency), AR	OMIM 120570	2.		21. CD59 (Membrane Attack Complex Inhibitor (CD59) deficiency, AR	OMIM 107271	21.	
3. C1QC (C1q deficiency), AR	OMIM 120575	3.		22. CFB (Factor B deficiency), AR	OMIM 615561	22.	
4. C1R (C1r deficiency), AR	OMIM 613785	4.		23. CFB (Factor B GOF), AD GOF	OMIM 612924	23.	
5. C1R (C1r Periodontal Ehlers-Danlos), AD GOF	OMIM 613785	5.		24. CFD (Factor D deficiency), AR	OMIM 134350	24.	
6. C1S (C1s deficiency), AR	OMIM 613785	6.		25. CFH (Factor H deficiency), AR or AD	OMIM 134370	25.	
7. C1S (C1s Periodontal Ehlers-Danlos), AD GOF	OMIM 613785	7.		26. CFHR1 (Factor H-related protein deficiencies), AR or AD	OMIM 134371	26.	
8. C2 (C2 deficiency), AR	OMIM 217000	8.		27. CFHR2 (Factor H-related protein deficiencies), AR or AD	OMIM 600889	27.	
9. C3 (C3 deficiency, LOF), AR	OMIM 120700	9.		28. CFHR3 (Factor H-related protein deficiencies), AR or AD	OMIM 605336	28.	
10. C3 (C3 GOF), AD GOF	OMIM 120700	10.		29. CFHR4 (Factor H-related protein deficiencies), AR or AD	OMIM 605337	29.	
11. C4A + C4B (Complete C4 deficiency), AR	OMIM 120810	11.		30. CFHR5 (Factor H-related protein deficiencies), AR or AD	OMIM 608593	30.	
12. C5 (C5 deficiency), AR	OMIM 120900	12.		31. CFI (Factor I deficiency), AR	OMIM 217030	31.	
13. C6 (C6 deficiency), AR	OMIM 217050	13.		32. CFP (Properdin deficiency), XL	OMIM 300383	32.	
14. C7 (C7 deficiency), AR	OMIM 217070	14.		33. FCN3 (Ficolin 3 deficiency), AR	OMIM 604973	33.	
15. C8A (C8α deficiency), AR	OMIM 120950	15.		34. MASP2 (MASP2 deficiency), AR	OMIM 605102	34.	
16. C8B (C8β deficiency), AR	OMIM 120960	16.		35. SERPING1 (C1 inhibitor deficiency), AD	OMIM 606860	35.	
17. C8G (C8γ deficiency), AR	OMIM 120930	17.		36. THBD (Thrombomodulin deficiency), AD	OMIM 188040	36.	
18. C9 (C9 deficiency), AR	OMIM 120940	18.		37. Other Complement Deficiencies: *Please also list in "Unspecified" section on Page 5		37.	
19. CD46 (Membrane Cofactor Protein (CD46) deficiency, AD	OMIM 120920	19.					

TABLE IX. BONE MARROW FAILURE

1.	ACD (DKCA6), AD	OMIM 616553	1.	23.	RAD51 (Fanconi anemia type R), AR	OMIM 617244	23.
2.	ACD (DKCB7), AR	OMIM 616553	2.	24.	RAD51C (Fanconi anemia type O), AR	OMIM 613390	24.
3.	BRCA1 (Fanconi anemia type S), AR	OMIM 617883	3.	25.	RFWD3 (Fanconi anemia type W), AR	OMIM 617784	25.
4.	BRCA2 (Fanconi anemia type D1), AR	OMIM 605724	4.	26.	RTEL1 (DKCA4), AD	OMIM 616373	26.
5.	BRIP1 (Fanconi anemia type J), AR	OMIM 609054	5.	27.	RTEL1 (DKCB5), AR	OMIM 615190	27.
6.	CTC1 (Coats plus syndrome), AR	OMIM 617053	6.	28.	SAMD9 (MIRAGE), AD GOF	OMIM 617053	28.
7.	DKC1 (DKCX1), XL	OMIM 305000	7.	29.	SAMD9L (Ataxia pancytopenia syndrome), AD GOF	OMIM 611170	29.
8.	ERCC4 (Fanconi anemia type Q), AR	OMIM 615272	8.	30.	SLX4 (Fanconi anemia type P), AR	OMIM 613951	30.
9.	FANCA (Fanconi anemia type A), AR	OMIM 227650	9.	31.	SRP72 (BMFS1 (SRP72 deficiency)), AD	OMIM 602122	31.
10.	FANCB (Fanconi anemia type B), XLR	OMIM 300514	10.	32.	STN1 (Coats plus syndrome), AR	OMIM 613129	32.
11.	FANCC (Fanconi anemia type C), AR	OMIM 227645	11.	33.	TERC (DKCA1), AD	OMIM 127550	33.
12.	FANCD2 (Fanconi anemia type D2), AR	OMIM 227646	12.	34.	TERT (DKCA2), AD	OMIM 187270	34.
13.	FANCE (Fanconi anemia type E), AR	OMIM 600901	13.	35.	TERT (DKCB4), AR	OMIM 613989	35.
14.	FANCF (Fanconi anemia type F), AR	OMIM 603467	14.	36.	TINF2 (DKCA3), AD	OMIM 604319	36.
15.	FANCI (Fanconi anemia type I), AR	OMIM 609053	15.	37.	TINF2 (DKCA5), AD	OMIM 268130	37.
16.	FANCL (Fanconi anemia type L), AR	OMIM 614083	16.	38.	TP53 (BMFS5), AD	OMIM 618165	38.
17.	FANCM (Fanconi anemia type M), AR	OMIM 618096	17.	39.	UBE2T (Fanconi anemia type T), AR	OMIM 616435	39.
18.	MAD2L2 (Fanconi anemia type V), AR	OMIM 617243	18.	40.	WRAP53 (DKCB3), AR	OMIM 613988	40.
19.	NOLA2 (DKCB2), AR	OMIM 613987	19.	41.	XRCC2 (Fanconi anemia type U), AR	OMIM 617247	41.
20.	NOLA3 (DKCB1), AR	OMIM 224230	20.	42.	XRCC9 (Fanconi anemia type G), AR	OMIM 614082	42.
21.	PALB2 (Fanconi anemia type N), AR	OMIM 610832	21.	43.	Other Phenocopies of Primary Immunodeficiencies:		43.
22.	PARN (DKCB6), AR	OMIM 616353	22.		*Please also list in "Unspecified" section on Page 5		

TABLE X. PHENOCOPIES OF INBORN ERRORS OF IMMUNITY

1.	AutoAB to IL-17 and/or IL-22 (CMC)	1.	8.	NLRP3 (Cryopyrinopathy, (Muckle-Wells/CINCA/NOMID-like syndrome))	8.
2.	AutoAB to Complement Factor H (Atypical hemolytic uremic syndrome)	2.	9.	NRAS (RALD), GOF	9.
3.	AutoAB to CI Inhibitor (Acquired anioedema)	3.	10.	TNFRSF6 (ALPS-SFAS)	10.
4.	AutoAB to GM-CSF (Pulmonary alveolar proteinosis)	4.	11.	STAT5B (Hypereosinophilic syndrome), GOF	11.
5.	AutoAB to IFNγ (Adult-onset immunodeficiency with susceptibility to mycobacteria)	5.	12.	AutoAB to various cytokines (Good syndrome))	12.
6.	AutoAB to IL-6 (Recurrent skin infection)	6.	13.	Other Phenocopies of Primary Immunodeficiencies:	13.
7.	KRAS (RALD), GOF	7.		*Please also list in "Unspecified" section on Page 5	

*OTHER PRIMARY IMMUNODEFICIENCY DISEASES - UNSPECIFIED

Please list the disease name, gene defect (if known), and/or other gene mutations and the corresponding number of patients in the correct deficiency classification

TABLE I. IMMUNODEFICIENCIES AFFECTING CELLULAR AND HUMORAL IMMUNITY	

TABLE VI. DEFECTS IN INTRINSIC AND INNATE IMMUNITY	

TABLE II. COMBINED IMMUNODEFICIENCIES WITH ASSOCIATED OR SYNDROMIC FEATURES	

TABLE VII. AUTOINFLAMMATORY DISORDERS	

TABLE III. PREDOMINANTLY ANTIBODY DEFICIENCIES	

TABLE VIII. COMPLEMENT DEFICIENCIES	

TABLE IV. DISEASES OF IMMUNE DYSREGULATION	

TABLE IX. BONE MARROW FAILURE	

TABLE V. CONGENITAL DEFECTS OF PHAGOCYTE NUMBER OR FUNCTION	

TABLE X. PHENOCOPIES OF INBORN ERRORS OF IMMUNITY	

PATIENT DEMOGRAPHICS

Please enter the approximate number of your patients in each of the age and gender categories listed below, if data is available.
This data will assist in developing a demographic assessment of PI within the Jeffrey Modell Centers Network.

AGE	
< 1 year	
1-4	
5-19	
20-39	
>40	

GENDER	
Male	
Female	

KEY TERMS

AD: Autosomal dominant inheritance
AR: Autosomal recessive inheritance
XL: X-linked inheritance
GOF: Gain-of-function mutation
LOF: Loss-of-function mutation

Please return to the Jeffrey Modell Foundation by email: jquinn@jmfworld.org or fax: 212-764-4180

© 2020 Jeffrey Modell Foundation

Tangye, S.G., Al-Herz, W., Bousfiha, A. et al. Human Inborn Errors of Immunity: 2019 Update on the Classification from the International Union of Immunological Societies Expert Committee. J Clin Immunol (2020).
https://doi.org/10.1007/s10875-019-00737-x

Online Mendelian Inheritance of Man (OMIM): https://www.omim.org
