# Supplementary material for: Growth in diagnosis and treatment of primary immunodeficiency within the global Jeffrey Modell Centers Network
Source: Allergy Asthma Clin Immunol. 2022 Mar 4;18:19. doi: 10.1186/s13223-022-00662-6 (PMC8896271; doi:10.1186/s13223-022-00662-6)
Supplement: Supplementary file 2 — Additional file 2: Table S1. Percentage of Missing Responses by Variable. The percentage of missing responses by variable, which ranges from 0.0 to 97.3% (n = 300). Variables correspond to the 2020–2021 Global Survey on Primary Immunodeficiencies. To note, for all variables other than demographics, a missing response was interpreted as a “zero” in that the physician did not have any patients having that particular condition or receiving that particular treatment. [file 13223_2022_662_MOESM2_ESM.pdf]

Table S1 Percentage of Missing Responses by Variable

Variable	% Missing Responses
Total # of patients being followed	0.0%
Total # of patients identified with a specific PI defect	0.3%
Total # of patients receiving IgG: IVIG - Clinic	5.7%
Total # of patients receiving IgG: IVIG - Home	37.3%
Total # of patients receiving IgG: SCIG	18.7%
Total # of patients receiving IgG: Other	70.0%
Total # of patients treated by Gene Therapy	32.0%
Total # of patients treated with PEG-ADA (Recombinant)	36.0%
Total # of patients treated by Transplant	20.7%
Donor type: MRD	52.0%
Donor type: MUD	53.3%
Donor type: mMUD	75.3%
Donor type: Parental Haplo	55.7%
Stem Cell Source: BM	51.7%
Stem Cell Source: PBSC	59.3%
Stem Cell Source: Cord	62.7%
Stem Cell Source: Other-specify	84.7%
ADA (Adenosine deaminase deficiency), AR	50.3%
AK2 (AK2 Defect), AR	83.0%
B2M (MHC class I deficiency), AR	93.3%
BCL10 (BCL10 deficiency), AR	94.3%
CARD 11 (CARD11 deficiency), AR LOF	85.0%
CD3D (CD3d deficiency), AR	84.0%
CD3E (CD3e deficiency), AR	90.7%
CD3G (CD3g deficiency), AR	92.7%
CD3Z (CD3z deficiency), AR	93.3%
CD40 (CD40 deficiency), AR	77.3%
CD40LG (CD40 ligand (CD154) deficiency), XL	46.0%
CD8A (CD8 deficiency), AR	89.0%
CIITA (MHC class II deficiency group A, B, C, D), AR	79.7%
CORO1A (Coronin-1A deficiency), AR	87.7%

DCLRE1C (Artemis deficiency), AR	71.7%
DOCK2 (DOCK2 deficiency), AR	91.0%
DOCK8 (DOCK8 deficiency), AR	56.7%
FCHO1 (FCHO1 deficiency), AR	94.7%
ICOS (ICOS deficiency), AR	86.7%
ICOSLG (ICOSL deficiency), AR	96.3%
IKBKB (IKBKB deficiency), AR	90.7%
IKZF1 (IKAROS deficiency), AD DN	91.3%
IL21 (IL-21 deficiency), AR	94.3%
IL21R (IL-21R deficiency), AR	87.0%
IL2RG (gc Deficiency, gc SCID, CD132 deficiency), XL	53.0%
IL7R (IL7Ra deficiency), AR	69.3%
ITK (ITK deficiency), AR	86.7%
JAK3 (JAK3 deficiency), AR	69.7%
LAT (LAT deficiency), AR	96.3%
LCK (LCK deficiency), AR	89.7%
LIG4 (DNA ligase IV deficiency), AR	83.0%
MALT1 (MALT1 deficiency), AR	85.7%
MAP3K14 (NIK deficiency), AR	94.3%
MSN (Moesin deficiency), XL	95.7%
NHEJ1 (Cernunnos/XLF deficiency), AR	89.7%
POLD 1 (Polymerase d deficiency), AR	96.0%
POLD 2 (Polymerase d deficiency), AR	96.0%
PRKDC (DNA PKcs deficiency), AR	87.0%
PTPRC (CD45 Deficiency), AR	89.3%
RAC2 (Activated RAC2 defect), AD GOF	93.7%
RAG1 (RAG deficiency), AR	52.3%
RAG2 (RAG deficiency), AR	67.0%
REL (c-Rel deficiency), AR	96.0%
RELA (RelA haploinsufficiency), AD	95.7%
RELB (RelB deficiency), AR	95.3%
RFX5 (MHC class II deficiency group A, B, C, D), AR	88.7%
RFXANK (MHC class II deficiency group A, B, C, D), AR	88.0%
RFXAP (MHC class II deficiency group A, B, C, D), AR	92.7%

RHOH (RHOH deficiency), AR	89.7%
STK4 (MST1 deficiency), AR	88.7%
TAP1 (MHC class I deficiency), AR	87.3%
TAP2 (MHC class I deficiency), AR	94.0%
TAPBP (MHC class I deficiency), AR	95.0%
TFRC (TFRC deficiency), AR	95.0%
TNFRSF4 (OX40 deficiency), AR	90.3%
TRAC (TCRa deficiency), AR	88.7%
ZAP70 (ZAP-70 combined mutations), AR (LOF/GOF)	94.7%
ZAP70 (ZAP-70 deficiency (ZAP70 LOF)), AR	77.7%
OTHER TABLE I	56.7%
11q23del (Chromosome 11q deletion syndrome(Jacobsen syndrome)), AD	87.0%
ARPC1B (Arp2/3-mediated filament branching defect), AR	92.3%
ATM (Ataxia-telangiectasia), AR	33.0%
BCL11B (BCL11B deficiency), AD	95.0%
BLM (Bloom Syndrome), AR	80.3%
CARD11 (CARD11 deficiency (heterozygous)), AD LOF	90.7%
CCBE1 (Hennekam-lymphangiectasia-lymphedema Syndrome), AR	93.7%
CDCA7 (ICF3), AR	95.7%
CHARGE Syndrome, unknown	80.7%
CHD7 (CHARGE Syndrome), AD	77.3%
Del10p13-p14 (Chromosome 10p13-p14 deletion syndrome), AD	96.3%
DiGeorge Syndrome, unknown	63.0%
DNMT3B (ICF1), AR	85.0%
EPG5 (EPG5 deficiency (Vici syndrome)), AR	93.7%
ERBB2IP (ERBIN deficiency), AD	97.0%
ERCC6L2 (Hebo deficiency), AR	97.0%
EXTL3 (EXTL3 deficiency), AR	95.7%
FAT4 (Hennekam-lymphangiectasia-lymphedema Syndrome), AR	96.3%
FOXN1 (FOXN1 haploinsufficiency), AD	93.7%
FOXN1 (Winged helix nude FOXN1 deficiency), AR	89.3%
GINS1 (GINS1 deficiency), AR	96.3%
HELLS (ICF4), AR	96.0%
IKBKB (EDA-ID due to GOF mutation), AD GOF	93.3%

IKBKG (EDA-ID due to NEMO/IKBKG deficiency), XL	75.0%
IL6R (IL6 receptor deficieny), AR	96.7%
IL6ST (IL6 signal transducer (IL6ST) deficiency), AR	95.7%
KDM6A (Kabuki syndrome), XL	93.0%
KMT2A (KMT2A deficiency (Wiedemann-Steiner syndrome)), AD	95.7%
KMT2D (Kabuki syndrome), AD	85.7%
LIG1 (Ligase I deficiency), AR	95.0%
MCM4 (MCM4 Deficiency), AR	89.7%
MTHFD1 (MTHFD1 deficiency), AR	95.3%
MYSM1 (MYSM1 deficiency), AR	96.0%
NBS1 (Nijmegen breakage syndrome), AR	75.3%
NFE2L2 (Activating de novo mutations in nuclear factor, erythroid 2-like)	97.0%
NFKBIA (EDA-ID due to IKBA GOF mutation), AD GOF	84.7%
NSMCE3 (NSMCE3 deficiency), AR	97.0%
ORAI1 (ORAI-I deficiency), AR	86.7%
PGM3 (PGM3 Deficiency), AR	91.3%
PMS2 (PMS2 Deficiency), AR	88.7%
PNP (PNP deficiency), AR	77.7%
POLE1 (FILS Syndrome), AR	88.7%
POLE2 (POLE2 deficiency), AR	97.0%
RBCK1 (HOIL1 deficiency), AR	90.3%
RMRP (Cartilage Hair Hypoplasia), AR	65.3%
RNF168 (RIDDLE Syndrome), AR	90.0%
RNF31 (HOIP deficiency), AR	95.0%
RNU4ATAC (MOPD1 deficiency (Roifman syndrome)), AR	90.3%
SEMA3E (CHARGE Syndrome), AD	94.3%
SKIV2L (Tricho-Hepato-Enteric Syndrome (THES)), AR	93.0%
SLC46A1 (SLC46A1/PCFT deficiency), AR	94.3%
SMARCAL1 (Schimke Immuno-osseous dysplasia), AR	78.3%
SP110 (VODI syndrome), AR	87.3%
SPINK5 (Comel-Netherton syndrome), AR	77.0%
STAT3 (AD-HIES STAT3 deficiency (Job syndrome)), AD LOF	37.7%
STAT5b (STAT5b deficiency), AD	94.7%
STAT5b (STAT5b deficiency), AR	89.0%

STIM1 (STIM-1 deficiency), AR	86.3%
TBX1 (DiGeorge, Chromosome 22q11.2 deletion syndrome), AD	41.3%
TBX1 (TBX1 Deficiency), AD	91.0%
TCN2 (Transcobalamin 2 deficiency), AR	94.7%
TGFBR1 (Loeys-Dietz syndrome (TGFBR deficiency)), AD	96.3%
TGFBR2 (Loeys-Dietz syndrome (TGFBR deficiency)), AD	97.0%
TTC37 (Tricho-Hepato-Enteric Syndrome (THES)), AR	87.7%
TTC7A (Immunodeficiency with multiple intestinal atresias), AR	84.3%
WAS (Wiskott-Aldrich syndrome (WAS LOF)), XL	32.3%
WIPF1 (WIP Deficiency), AR	89.7%
ZBTB24 (ICF2), AR	87.0%
ZNF341 (ZNF341 deficiency AR-HIES), AR	96.0%
OTHER TABLE II	68.3%
AICDA (AID deficiency), AR	76.3%
TNFRSF13C (BAFF receptor deficiency), AR	88.0%
BLNK (BLNK deficiency), AR	86.0%
BTK (BTK deficiency, XLA) XL	23.3%
CARD11 (CARD11 GOF), AD GOF	89.3%
CD19 (CD19 deficiency), AR	86.3%
CD20 (CD20 deficiency), AR	90.3%
CD21 (CD21 deficiency), AR	89.7%
CD81 (CD81 deficiency), AR	90.7%
Transient hypogammaglobulinemia of Infancy	43.0%
CD79A (Iga deficiency), AR	85.0%
CD79B (Igb deficiency), AR	89.7%
IgG subclass deficiency with IgA deficiency	57.3%
Isolated IgG subclass deficiency	51.7%
Ig heavy chain mutations and deletions, AR	88.7%
IGKC (kappa chain deficiency), AR	95.0%
INO80 (INO80 deficiency), AR	94.7%
IGHM (m heavy chain deficiency), AR	87.7%
MOGS (MOGS deficiency), AR	95.0%
MSH6 (MSH6 deficiency), AR	94.3%
NFKB1 (NFKB1 deficiency), AD	84.7%

PIK3R1 (Activated p110d syndrome (APDS1)), AD	76.3%
PIK3R1 (p85 deficiency), AR	93.7%
PIK3CD GOF (Activated p110d syndrome (APDS2)), AD	67.7%
Specific antibody deficiency (normal Ig and B cells)	49.3%
ATP6AP1 (ATP6AP1 deficiency), XL	96.7%
TRNT1 (TRNT1 deficiency), AR	92.3%
TNFS12 (TWEAK deficiency), AD	90.0%
UNG (UNG deficiency), AR	88.7%
IGLL1 (l5 deficiency), AR	89.0%
PIK3CD (p110d deficiency), AR	92.3%
TCF3 (E47 transcription factor deficiency), AD	88.3%
TCF3 (E47 transcription factor deficiency), AR	96.3%
SLC39A7 (ZIP7 deficiency), AR	97.3%
TOP2B (Hoffman syndrome/TOP2B deficiency), AD	96.7%
CVID, unknown	12.3%
PTEN (PTEN deficiency (LOF)), AD	92.3%
TNFRSF13B (TACI deficiency), AR or AD	68.7%
NFKB2 (NFKB2 deficiency), AD	75.7%
IKZF1 (IKAROS deficiency), AD	91.3%
IRF2BP2 (IRF2BP2 deficiency), AD	95.3%
ARHGEF1 (ARHGEF1 deficiency), AR	97.0%
SH3KBP1 (SH3KBP1 (CIN85) deficiency), XL	97.0%
SEC61A1 (SEC61A1 deficiency), AD	96.3%
RAC2 (RAC2 deficiency), AR	95.0%
AICDA (AID deficiency), AD	94.3%
Selective IgA deficiency	44.0%
Selective IgM deficiency	83.0%
OTHER TABLE III	66.0%
AIRE (APECED (APS-1), AR or AD	62.7%
AP3B1 (Hermansky-Pudlak syndrome type 2), AR	85.0%
AP3D1 (Hermansky-Pudlak syndrome type 10), AR	96.7%
BACH2 (BACH2 deficiency), AD	94.3%
CARMIL2 (RLTPR deficiency), AR	91.3%
CASP10 (ALPS-Caspase 10), AD	83.7%

CASP8 (ALPS-Caspase 8), AR	87.3%
CD27 (CD27 deficiency), AR	88.7%
CD70 (CD70 deficiency), AR	95.3%
CTLA4 (CTLA4 haploinsufficiency (ALPS-V)), AD	71.7%
CTPS1 (CTPS1 deficiency), AR	93.7%
DEF6 (DEF6 deficiency), AR	97.0%
FAAP24 (FAAP24 deficiency), AR	97.0%
FADD (FADD deficiency), AR	90.3%
FERMT1 (FERMT1 deficiency), AR	96.7%
FOXP3 (IPEX syndrome), XL	61.7%
IL10 (IL-10 deficiency), AR	87.0%
IL10RA (IL-10R deficiency), AR	83.3%
IL10RB (IL-10R deficiency), AR	86.0%
IL2RA (CD25 deficiency), AR	86.3%
IL2RB (CD122 deficiency), AR	96.3%
ITCH (ITCH deficiency), AR	89.3%
JAK1 (JAK1 GOF), AD GOF	95.3%
LRBA (LRBA deficiency), AR	68.3%
LYST (Chediak-Higashi syndrome), AR	71.7%
MAGT1 (XMEN), XL	83.7%
NFAT5 (NFAT5 haploinsufficiency), AD	95.0%
PEPD (Prolidase deficiency), AR	95.0%
PRF1 (Perforin deficiency (FHL2)), AR	76.7%
PRKCD (PRKCD deficiency), AR	87.7%
RAB27A (Griscelli syndrome type 2), AR	80.0%
RASGRP1 (RASDRP1 deficiency), AR	94.7%
RIPK1 (RIPK1), AR	95.0%
SH2D1A (SAP deficiency (XLP1), XL	65.3%
SLC7A7 (SLC7A7 deficiency), AR	96.0%
STAT3 (STAT3 GOF mutation), AD GOF	74.7%
STX11 (Syntaxin 11 deficieny (FHL4)), AR	88.0%
STXBP2 (STXBP2/Munc18-2 deficiency (FHL5)), AR or AD	81.3%
TGFB1 (TGFB1 deficiency), AR	96.7%
TNFRSF6 (ALPS-FAS), AD or AR	66.7%

TNFRSF9 (CD137 deficiency (41BB)), AR	95.7%
TNFSF6 (ALPS-FASLG), AR	84.7%
TPP2 (Tripeptidyl-peptidase II deficiency), AR	94.7%
UNC13D (UNC13D/Munc12-4 deficiency (FHL3)), AR	75.0%
XIAP (XIAP deficiency (XLP2), XL	66.0%
OTHER TABLE IV	73.7%
ACTB (b-Actin deficiency), AD	89.7%
CEBPE (Specific granule deficiency), AR	88.3%
CFTR (Cystic fibrosis), AR	91.7%
CLBP (3-Methylglutaconic aciduria), AR	95.0%
CSF2RA (Pulmonary alveolar proteinosis), XL	89.3%
CSF2RB (Pulmonary alveolar proteinosis), AR	96.7%
CSF3R (G-CSF receptor deficiency), AR	94.0%
CTSC (Papillon-Lefèvre syndrome), AR	85.7%
CYBA (CGD), AR	67.7%
CYBB (X-linked CGD (gp91 phox)), XL	35.3%
CYBC1 (CGD), AR	94.0%
DNAJC21 (Shwachman-Diamond Syndrome), AR	94.7%
EFL1 (Shwachman-Diamond Syndrome), AR	95.0%
ELANE (Elastase deficiency (Severe congenital neutropenia [SCN] 1)), AD	56.3%
FERMT3 (Leukocyte adhesion deficiency type 3 (LAD3)), AR	85.0%
FPR1 (Localized juvenile periodontitis), AR	90.0%
G6PC3 (G6PC3 deficiency (SCN4)), AR	83.7%
G6PD (G6PD deficiency class I), XL	94.3%
G6PT1 (Glycogen storage disease type 1b), AR	83.3%
GATA2 (GATA2 deficiency), AD	69.3%
GFI1 (GFI1 deficiency (SCN2)), AD	90.3%
HAX1 (HAX1 deficiency (Kostmann Disease (SCN3))), AR	83.0%
HYOU1 (HYOU1 deficiency), AR	96.3%
ITGB2 (Leukocyte adhesion deficiency type 1 (LAD1)), AR	69.7%
JAGN1 (JAGN1 deficiency), AR	86.0%
LAMTOR2 (P14/LAMTOR2 deficiency), AR	90.7%
MKL1 (Neutropenia with combined immune deficiency), AR	95.7%
NCF1 (CGD), AR	66.7%

NCF2 (CGD), AR	79.0%
NCF4 (CGD), AR	85.7%
RAC2 (Rac2 deficiency), AD LOF	88.7%
SBDS (Shwachman-Diamond Syndrome), AR	75.7%
SLC35C1 (Leukocyte adhesion deficiency type 2 (LAD2)), AR	86.7%
SMARCD2 (SMARCD2 deficiency), AR	96.3%
SRP54 (SRP54 deficiency), AD	95.3%
TAZ (Barth syndrome), XL	86.3%
USB1 (Clericuzio syndrome), AR	95.3%
VPS13B (Cohen syndrome), AR	87.0%
VPS45 (VPS45 deficiency (SCN5)), AR	87.7%
WAS (X-linked neutropenia/myelodysplasia), XL GOF	83.3%
WDR1 (WDR1 deficiency), AR	96.7%
OTHER TABLE V	71.7%
APOL1 (Trypanosomiasis), AD	90.7%
CARD9 (CARD9 deficiency), AR	86.3%
CIB1 (CIB1 deficiency (HPV))	96.7%
CLCN7 (Osteopetrosis), AR	95.7%
CXCR4 (WHIM Syndrome (HPV)), AD GOF	74.7%
CYBB (Macrophage gp91 phox deficiency (MSMD)), XL	87.7%
DBR1 (DBR1 deficiency (HSE)), AR	97.0%
FCGR3A (CD16 deficiency), AR	90.0%
HMOX (Isolated congenital asplenia (ICA)), AR	95.0%
IFIH1 (MDA5 deficiency), AR LOF	95.7%
IFNAR1 (IFNAR1 deficiency), AR	95.7%
IFNAR2 (IFNAR2 deficiency), AR	96.3%
IFNGR1 (IFN-γ receptor 1 deficiency (MSMD)), AD	83.3%
IFNGR1 (IFN-γ receptor 1 deficiency (MSMD)), AR	89.0%
IFNGR2 (IFN-γ receptor 2 deficiency (MSMD)), AR	85.0%
IL12B (IL-12p40 (IL-12 and IL-23) deficiency (MSMD)), AR	86.7%
IL12RB1 (IL-12 and IL-23 receptor b1 chain deficiency (MSMD)), AR	76.3%
IL12RB2 (IL-12Rb2 deficiency (MSMD)), AR	95.0%
IL17F (IL-17F deficiency (CMC)), AD	86.3%
IL17RA (IL-17RA deficiency (CMC)), AR	86.7%

IL17RC (IL-17RC deficiency (CMC)), AR	94.3%
IL18BP (IL-18BP deficiency), AR	97.0%
IL23R (IL-23R deficiency (MSMD)), AR	97.0%
IRAK1 (IRAK1 deficiency), XL	97.0%
IRAK4 (IRAK4 deficiency), AR	81.3%
IRF3 (IRF3 deficiency (HSE)), AD	95.3%
IRF4 (IRF4 haploinsufficiency), AD	96.7%
IRF7 (IRF7 deficiency), AR	95.0%
IRF8 (IRF8 defiency (MSMD)), AD	89.0%
IRF8 (IRF8 defiency (MSMD)), AR	96.0%
IRF9 (IRF9 deficiency), AR	96.7%
ISG15 (ISG15 deficiency (MSMD)), AR	94.7%
JAK1 (JAK1 deficiency (MSMD)), AR LOF	96.7%
MYD88 (MyD88 deficiency), AR	85.7%
NBAS (Acute liver failure due to NBAS deficiency), AR	94.7%
NCSTN (Hidradenitis suppurativa), AD	96.7%
OSTM1 (Osteopetrosis), AR	96.3%
PLEKHM1 (Osteopetrosis), AR	96.7%
POLR3A (RNA polymerase III deficiency), AD	97.0%
POLR3C (RNA polymerase III deficiency), AD	97.0%
POLR3F (RNA polumerase III deficiency), AD	97.0%
PSEN (Hidradenitis suppurativa), AD	96.3%
PSENEN (Hidradenitis suppurativa), AD	96.7%
RANBP2 (Acute necrotizing encephalopathy), AR	96.0%
RORC (RORgt deficiency (MSMD)), AR	95.3%
RPSA (Isolated congenital asplenia (ICA)), AD	89.0%
SNX10 (Osteopetrosis), AR	96.7%
SPPL2A (SPPL2a deficiency (MSMD)), AR	96.7%
STAT1 (STAT1 deficiency (MSMD)), AD LOF	82.3%
STAT1 (STAT1 deficiency), AR LOF	90.3%
STAT1 (STAT1 GOF (CMC)), AD GOF	62.0%
STAT2 (STAT2 deficiency), AR	88.7%
TBK1 (TBK1 deficiency (HSE)), AD	89.3%
TCIRG1 (Osteopetrosis), AR	95.7%

TICAM1 (TRIF deficiency (HSE)), AD or AR	89.3%
TIRAP (TIRAP deficiency), AR	96.7%
TLR3 (TLR3 deficiency (HSE)), AD or AR	87.3%
TMC6 (EVER1 deficiency (HPV)), AR	89.3%
TMC8 (EVER2 deficiency (HPV))	89.7%
TNFRSF11A (Osteopetrosis), AR	97.0%
TNFSF11 (Osteopetrosis), AR	97.0%
TRAF3 (TRAF3 deficiency (HSE)), AD	89.7%
TRAF3IP2 (ACT1 deficiency), AR	89.3%
TYK2 (P1104A TYK2 homozygosity (MSMD)), AR	96.0%
TYK2 (Tyk2 deficiency (MSMD)), AR	88.0%
UNC93B1 (UNC93B1 deficiency (HSE)), AR	88.7%
NK Cell Deficiency	82.7%
OTHER TABLE VI	74.0%
ADAM17 (ADAM17 deficiency), AR	94.3%
IFIH1 (AGS7), AD GOF	89.3%
SH3BP2 (Cherubism), AD	90.0%
NLRP3 (Familial cold autoinflammatory syndrome 1), AD GOF	89.0%
DNASE2 (DNAse II deficiency), AR	96.7%
MEFV (Familial Mediterranean fever), AD	61.7%
TNFRSF1A (TNF receptor-associated periodic syndrome (TRAPS)), AD	70.0%
ADAR1 (ADAR1 deficiency, AGS6)), AR	93.3%
OAS1 (OAS1 deficiency), AD GOF	97.0%
ACP5 (SPENCD), AR	89.0%
ALP1 (ALP1 deficiency), AR	97.0%
NOD2 (Blau syndrome), AD	80.3%
NLRP3 (Muckle-Wells syndrome), AD GOF	81.0%
MEFV (Familial Mediterranean fever), AR LOF	85.3%
PSMB8 (CANDLE), AR and AD	87.0%
MVK (Mevalonate kinase deficiency (Hyper IgD syndrome), AR	66.3%
POLA1 (X-linked reticulate pigmentary disorder), XL	96.0%
COPA (COPA defect), AD	91.3%
CARD14 (CAMPS (CARD14 mediated psoriasis)), AD	89.0%
SLC29A3 (SLC29A3 mutation), AR	89.7%

PSTPIP1 (PAPA syndrome, hyperzincemia, & hypercalprotectinemia), AD	83.3%
RNASEH2A (RNASEH2A deficiency (AGS4)), AR	94.3%
TRIM22 (TRIM22), AR	97.0%
TREX1 (TREX1 deficiency (AGS1)), AR	93.7%
SAMHD1 (SAMHD1 deficiency (AGS5)), AR	89.3%
USP18 (USP18 deficiency), AR	96.7%
NLRP3 (NOMID or CINCA), AD GOF	80.0%
ADA2 (ADA2 deficiency), AR	82.0%
LPIN2 (Majeed syndrome), AR	89.3%
PSMG2 (CANDLE), AR	96.0%
RNASEH2B (RNASEH2B deficiency (AGS2)), AR	93.3%
RNASEH2C (RNASEH2C deficiency (AGS3)), AR	95.3%
NLRP12 (Familial cold autoinflammatory syndrome 2), AD GOF	83.7%
TMEM173 (SAVI), AR	90.3%
IL1RN (DIRA), AR	88.3%
IL36RN (DITRA), AR	90.3%
DNASE1L3 (DNASE1L3 deficiency), AR	96.7%
PLCG2 (FCAS3, or APLAID), AD GOF	90.7%
PLCG2 (PLAID), AD GOF	87.7%
NLRP1 (NLRP1 GOF), AD GOF	96.0%
OTULIN (Otulipenia/ORAS), AR	96.3%
AP1S3 (AP1S3 deficiency), AR	97.0%
NLRC4 (NLRC4-MAS), AD GOF	95.3%
NLRC4 (Familial cold autoinflammatory syndrome 4), AD GOF	93.7%
TNFAIP (A20 deficiency), AD	94.3%
NLRP1 (NLRP1 deficiency), AR	97.0%
HAVCR2 (TIM3 deficiency), AR	97.0%
OTHER TABLE VII	73.0%
C1QA (C1q deficiency), AR	71.0%
C1QB (C1q deficiency), AR	92.3%
C1QC (C1q deficiency), AR	89.7%
C1R (C1r deficiency), AR	96.3%
C1R (C1r Periodontal Ehlers-Danlos), AD GOF	97.0%
C1S (C1s deficiency), AR	89.7%

C1S (C1s Periodontal Ehlers-Danlos), AD GOF	97.0%
C2 (C2 deficiency), AR	64.3%
C3 (C3 deficiency, LOF), AR	82.0%
C3 (C3 GOF), AD GOF	93.7%
C4A + C4B (Complete C4 deficiency), AR	79.0%
C5 (C5 deficiency), AR	85.7%
C6 (C6 deficiency), AR	86.0%
C7 (C7 deficiency), AR	82.0%
C8A (C8a deficiency), AR	86.7%
C8B (C8b deficiency), AR	96.3%
C8G (C8g deficiency), AR	96.3%
C9 (C9 deficiency), AR	88.3%
CD46 (Membrane Cofactor Protein (CD46) deficiency, AD	89.3%
CD55 (CD55 deficiency (CHAPEL disease)), AR	94.7%
CD59 (Membrane Attack Complex Inhibitor (CD59) deficiency, AR	90.3%
CFB (Factor B deficiency), AR	96.7%
CFB (Factor B GOF), AD GOF	90.0%
CFD (Factor D deficiency), AR	90.3%
CFH (Factor H deficiency), AR or AD	85.0%
CFHR1 (Factor H-related protein deficiencies), AR or AD	90.0%
CFHR2 (Factor H-related protein deficiencies), AR or AD	97.0%
CFHR3 (Factor H-related protein deficiencies), AR or AD	96.7%
CFHR4 (Factor H-related protein deficiencies), AR or AD	97.0%
CFHR5 (Factor H-related protein deficiencies), AR or AD	96.7%
CFI (Factor I deficiency), AR	84.0%
CFP (Properdin deficiency), XL	84.7%
FCN3 (Ficolin 3 deficiency), AR	89.3%
MASP2 (MASP2 deficiency), AR	89.7%
SERPING1 (C1 inhibitor deficiency), AD	83.3%
THBD (Thrombomodulin deficiency), AD	90.0%
OTHER TABLE VIII	80.0%
ACD (DKCA6), AD	96.7%
ACD (DKCB7), AR	97.0%
BRCA1 (Fanconi anemia type S), AR	97.0%

BRCA2 (Fanconi anemia type D1), AR	97.0%
BRIP1 (Fanconi anemia type J), AR	97.3%
CTC1 (Coats plus syndrome), AR	97.3%
DKC1 (DKCX1), XL	82.3%
ERCC4 (Fanconi anemia type Q), AR	96.7%
FANCA (Fanconi anemia type A), AR	94.0%
FANCB (Fanconi anemia type B), XLR	96.7%
FANCC (Fanconi anemia type C), AR	97.0%
FANCD2 (Fanconi anemia type D2), AR	97.0%
FANCE (Fanconi anemia type E), AR	97.0%
FANCF (Fanconi anemia type F), AR	97.3%
FANCI (Fanconi anemia type I), AR	97.3%
FANCL (Fanconi anemia type L), AR	97.3%
FANCM (Fanconi anemia type M), AR	97.3%
MAD2L2 (Fanconi anemia type V), AR	97.3%
NOLA2 (DKCB2), AR	90.3%
NOLA3 (DKCB1), AR	97.0%
PALB2 (Fanconi anemia type N), AR	97.0%
PARN (DKCB6), AR	97.0%
RAD51 (Fanconi anemia type R), AR	96.7%
RAD51C (Fanconi anemia type O), AR	97.3%
RFWD3 (Fanconi anemia type W), AR	97.3%
RTEL1 (DKCA4), AD	94.3%
RTEL1 (DKCB5), AR	95.7%
SAMD9 (MIRAGE), AD GOF	92.7%
SAMD9L (Ataxia pancytopenia syndrome), AD GOF	95.3%
SLX4 (Fanconi anemia type P), AR	97.3%
SRP72 (BMFS1 (SRP72 deficiency)), AD	97.3%
STN1 (Coats plus syndrome), AR	97.3%
TERC (DKCA1), AD	86.3%
TERT (DKCA2), AD	92.3%
TERT (DKCB4), AR	96.7%
TINF2 (DKCA3), AD	95.7%
TINF2 (DKCA5), AD	96.7%

TP53 (BMFS5), AD	97.0%
UBE2T (Fanconi anemia type T), AR	97.3%
WRAP53 (DKCB3), AR	97.0%
XRCC2 (Fanconi anemia type U), AR	97.3%
XRCC9 (Fanconi anemia type G), AR	97.3%
OTHER TABLE IX	94.7%
AutoAB to IL-17 and/or IL-22 (CMC)	93.7%
AutoAB to Complement Factor H (Atypical hemolytic uremic syndrome)	94.0%
AutoAB to CI Inhibitor (Acquired anioedema)	92.0%
AutoAB to GM-CSF (Pulmonary alveolar proteinosis)	94.7%
AutoAB to IFNg (Adult-onset immunodeficiency with susceptibility to mycobacteria)	91.3%
AutoAB to IL-6 (Recurrent skin infection)	95.3%
KRAS (RALD), GOF	88.7%
NLRP3 (Cryopyrinopathy, (Muckle-Wells/CINCA/NOMID-like syndrome))	93.3%
NRAS (RALD), GOF	88.0%
TNFRSF6 (ALPS-SFAS)	94.3%
STAT5B (Hypereosinophilic syndrome), GOF	96.3%
AutoAB to various cytokines (Good syndrome))	93.0%
OTHER TABLE X	95.3%
Age < 1 year	62.3%
Age 1-4 years	60.3%
Age 5-19 years	59.7%
Age 20-39 years	62.7%
Age 40+ years	66.3%
Male	56.0%
Female	55.7%

The percentage of missing responses by variable, which ranges from 0.0% to 97.3% (n=300). Variables correspond to the 2020-2021 Global Survey on Primary Immunodeficiencies. To note, for all variables other than demographics, a missing response was interpreted as a "zero" in that the physician did not have any patients having that particular condition or receiving that particular treatment.
